# Blood flow in the human cerebral cortex: Large-scale pial vascularization and 1D simulation

**DOI:** 10.1371/journal.pcbi.1013459

**Published:** 2025-09-08

**Authors:** Eduardo G. Zilves, Lucas O. Müller, Gonzalo D. M. Talou, Vladimir Hachinski, J. David Spence, Pablo J. Blanco

**Affiliations:** 1 Department of Mathematical and Computational Methods, National Laboratory for Scientific Computing, Petrópolis, Brazil; 2 Department of Mathematics, University of Trento, Trento, Italy; 3 Auckland Bioengineering Institute, University of Auckland, Auckland, New Zealand; 4 Department of Clinical Neurological Sciences, Western University, London, Canada; 5 Stroke Prevention and Atherosclerosis Research Centre, Robarts Research Institute, Western University, London, Canada; Centre National de la Recherche Scientifique, FRANCE

## Abstract

Understanding cerebral circulation is crucial for early diagnosis and patient-oriented therapies for brain conditions. However, blood flow simulations at the organ scale have been limited. This work introduces a framework for modeling extensive vascular networks in the human cerebral cortex and conducting pulsatile blood flow simulations. Using a patient-specific cerebral geometry, we applied a parallelized adaptive constrained constructive optimization algorithm to create a comprehensive pial vascular network in the left hemisphere, starting from the main cerebral arteries. The resulting network included over 75 000, 103 000, and 55 000 vessels for the anterior, middle, and posterior territories, respectively. Pial vessel diameters featured a median [interquartile range, IQR] value of 62.8[49.3,89.3]μm. We integrated the pial vascular network model with the Anatomically-Detailed Arterial Network (ADAN) model to conduct one-dimensional (1D) blood flow simulations under normotensive and hypertensive conditions. Viscoelastic dissipation proved to be a key ingredient in the characterization of the hemodynamic environments in the pial circulation. In the normotensive scenario, mean blood pressure in the pial vessels resulted in a median [IQR] value of 56.7[49.8,63.5]mmHg. The flow pulsatility index and its corresponding damping factor were effective descriptors of the hypertensive state. The median [IQR] pulsatility index in the normotensive state was 0.39[0.38,0.40], and in hypertension it increased up to 0.84[0.83,0.85], while its corresponding damping factor in the normotensive state was 2.07[1.78,2.48], and in the hypertensive state it was reduced to 1.20[1.16,1.39]. We observed large regional pressure gradients in terminal vessels, with pressure levels ranging from 50mmHg in normotension to 70mmHg in hypertension. Additionally, the pulsatility index at terminal vessels increased with distance from the Circle of Willis in the hypertensive case, contrasting with the decreasing pattern seen in normotension. This approach provides a unique characterization of hemodynamics in the pial vascular network of the human cerebral cortex, paving the way for research into microcirculatory environments, the link between hemodynamics and neural function, and their roles in conditions like stroke and dementia.

## Author summary

Understanding cerebral circulation is key to creating strategies for early diagnosis of brain conditions and patient-oriented therapeutic strategies. While cerebral circulation spans several spatial scales, there has been limited progress in conducting blood flow simulations at the scale of the entire organ. To fill this gap, we first synthesized the entire pial vascular network of the human cerebral cortex, maintaining consistency with the complex geometry of the brain. Second, we performed blood flow simulations to analyze the hemodynamic environments encountered across the different regions over the cerebral cortex. Results reported in this work help us to understand circulatory conditions in normal and hypertensive scenarios, something essential to investigate the relation between hemodynamics and neurovascular and neurodegenerative diseases such as stroke and dementia.

## Introduction

While cardiovascular diseases continue to lead the causes of death worldwide, cerebrovascular diseases have increased their incidence by over 70% from 1990 to 2019, becoming the second leading cause of death in the elderly population [1]. In turn, Alzheimer’s disease and other related dementias have also increased by over 140% between 1990 and 2019 [2]. Moreover, as the population continues to get older and life expectancy increases, the prevalence of these diseases is expected to rise even more. Currently, proper assessment of cerebrovascular conditions can be achieved by different medical imaging techniques, such as computed tomography and magnetic resonance [3]. In contrast, alternative approaches seek specific biomarkers related to brain injury, such as proteins, enzymes, and hormones [4], targeting the early diagnosis of pathologies. In this regard, increasing our understanding of the mechanisms underlying brain injury, caused either by ischemia or by neurodegenerative diseases, is of the utmost importance to achieving early diagnoses, as well as defining patient-specific therapeutic strategies.

Brain function is a consequence of the reciprocal interaction among the different hierarchical layers of brain organization, from gene expression to molecular scales, from cellular metabolism to neural activity, and from there to hemodynamic and vascular adaptations, in both physiological and pathological conditions [5]. Functional brain units are used to describe the brain’s inner workings within graph theory at an observable spatial scale [6]. These units are interconnected, and the strength in functional connectivity has a significant spatial correlation with metabolic markers and, therefore, with regional cerebral blood flow [7]. A similar organization has been acknowledged at the smallest scales, where the concept of the neurovascular unit brought new ways of interpreting the two-way coupling between neural activity and hemodynamics, which remains to be fully elucidated [8]. In turn, the neurovasculome concept emerged as a paradigm to understand brain function, providing a holistic framework to interpret, at the whole-organ scale, the pathological disruption of fundamental mechanisms, and its connection to neurovascular and neurodegenerative diseases [9]. The assessment of these multi-scale and multi-system interactions remains out of the scope of any current technology, and given these technological limitations, computational models have emerged as powerful instruments in this field of research as complementary tools to speed up research.

Blood flow modeling and numerical simulations have produced a significant advancement in the field of biomedical engineering, and translational research continues to have an impact on medical practice. The fusion of medical imaging and computational fluid dynamics has made possible the simulation of coronary blood flow phenomena in patient-specific geometries [10–12], the study of hemodynamics after Fontan procedures [13], the assessment of organ-specific blood pressure and flow conditions under physiological and pathophysiological assumptions, such as the liver [14], the kidney [15], and the brain [16,17], and the planning of surgical procedures and implantation of medical devices [18–20]. In the context of the present work, computer simulations are valuable tools for testing hypotheses regarding the complex interactions between neural activity and hemodynamics, their connections to brain metabolism, and cerebral blood flow regulation. Computational models can shed light on the fundamental principles of brain physiology and pathophysiology at different spatial and temporal scales, from the fast neuronal signaling, the relation to intra-parenchymal arterioles adaptations, the consequences on the vascular autoregulation mechanisms, and ultimately into the systemic blood flow dynamics at the organ scale. Also, computer simulations can be employed to investigate and develop patient-specific therapeutic strategies for brain disorders, targeting, for example, blood pressure control as a surrogate of brain health.

Various approaches have focused on modeling cerebral blood flow with different degrees of detail. At the smallest scales, Reichold et al. [21] employed tomographic microscopy data to reconstruct the vasculature within a prototypical region of the rat cerebral cortex and analyzed hemodynamics by simulating blood flow and oxygen concentration under steady-state conditions. Similarly, Gould et al. [22] employed large vascular networks within a representative vascular unit of cortical tissue to study the pressure drop across the entire spectrum of vessel sizes within the vascular network, also considering the steady-state assumption. In Lininger et al. [23], the authors synthesized the vascular network of the entire mouse brain, while in studies by Hartung et al. [24], closure criteria were employed to connect, through capillary beds, both arterial and venous networks in these mouse brain models. Using a multi-scale approach, Padmos et al. [25] coupled pulsatile 1D models of the large cerebral vessels with the brain tissue modeled as a porous medium in a steady state condition, and compared healthy and ischemic conditions caused by a vessel obstruction. Another multi-scale approach employing 1D and porous media models was reported by Koppl et al. [26]. In that work, the authors explored the gas exchange between a vascular network [21] and its surrounding tissue. In turn, whole-brain perfusion was modeled using a porous media model by Jósza et al. [27], where the impact of occlusions was assessed. A more realistic model was proposed by Otani et al. [28], where patient-specific imaging was used to build a model of the main vascular branches and of the pial surface, from which synthetic vascular networks for the mid-sized pial vessels were constructed and coupled to a structured representation of the remaining pial vascular network. Steady-state simulations were conducted to assess the effect of collateral vessels in defining the ischemic regions affected by arterial occlusions. In Linninger et al. [29] the authors proposed a methodology to extend arterial and venous vascular networks from patient-specific medical images, providing a full-organ characterization of the cortical vascular network in the human brain. Steady-state compartmental model simulations, and timedependent tracer concentration simulations were reported, simulating magnetic resonance metrics.

Moreover, modeling instruments can be important to provide mechanistic support to medical hypotheses and observations. For example, the location of the maximal damage due to hypertension (basal ganglia, thalamus and brain stem) was explained on the bases of the evolution of the brain’s blood supply [30]. More recently the concept was extended to explain deep white matter vasculature in terms of differential regional blood pressures [31], where a new paradigm was proposed based on the coexistence of low-pressure and high-pressure brain sub-systems. This new interpretation of the brain circulation has been termed the ambibaric brain theory and would help to pave the way towards understanding not only pathophysiological conditions, such as the hypotensive conditions encountered in hypertensive patients, and *β*-amyloid deposition, but also to establish novel diagnostic and treatment strategies. In line with the ambibaric brain theory, Blanco et al. [32] investigated pressure gradients in the brain and found significant differences in blood pressure between centrencephalic areas and cortical regions. However, the model proposed in the aforementioned study did not take into account the specificities of the pial surface where supplied vascular territories are defined. As a way to increase the predictive and descriptive capabilities of the model developed in that work, in the present study we propose an approach to construct an anatomically consistent pial vascular network spanning the entire cerebral cortex over the left hemisphere of the human brain. This is achieved by expanding the cerebral vasculature defined by pre-existing cerebral vessels down to the spatial scale defined by those vessels located immediately before the the penetrating arterioles over the pial surface. The pial surface geometry is obtained from magnetic resonance imaging, and the cerebral vessels used as an initial vascular network are those already available in the ADAN model [33], although patient-specific vessel centerlines could be employed if high resolution angiographic MRI data were available. The ADAN vascular network is first registered on top of the patient’s pial surface and is then expanded to cover the entire left hemisphere using a Constrained Constructive Optimization (CCO)-based algorithm [34–37]. Scaling up the vascular generation for the whole left hemisphere pial surface has been possible through the use of a parallel adaptive version of the CCO method, called PDCCO [38,39]. The resulting pial network spans over $230\;\cdot \;10^{3}$ segments of pial vessels, targeting quantities comparable with the vascular density reported in vivo in the literature, of around 1.2 penetrating arterioles per square millimeter [40]. In addition, pulsatile blood flow simulations employing the 1D blood flow theory [41] are used to investigate the hemodynamics with a high level of detail around the different cortical regions. Blood pressure gradients and blood flow distribution are evaluated in normotensive and hypertensive conditions. Flow- and pressure-based damping factors which are related to the transfer of energy to distal locations are also reported and discussed.

Thus, the present work introduces two main novel aspects: (i) the creation of a comprehensive model of the pial arterial blood vessels featuring anatomical consistency with a patient-specific cerebrum geometry, and (ii) the use of the 1D blood flow theory to conduct pulsatile simulations in these massive vascular networks. Such simulations can serve not only to improve our understanding of essential aspects of cerebral blood flow dynamics but also as reference solutions in the future to develop and validate simplified blood flow models.

## Materials and methods

This section details first the procedure to build the vascular networks for the three vascular territories in the human cerebral cortex, from the medical image processing to the multi-stage automatic vascular generation. Then, the 1D blood flow model is described, together with the scenarios of interest.

### Patient-specific cerebrum

A young-adult male participant was scanned using a 3T MRI scanner (SIGNA Premier; General Electric Healthcare, MI, USA) using an AIR 48-channel head coil, and provided written informed consent. Concerning MR scan data and associated data, in line with the approved ethics documentation from this study, endorsed by the New Zealand Health and Disability Ethics Committees (NZ HDEC), and in accordance with the Indigenous and community engagement policies, non-identifiable data are available upon request and subsequent approval by the Mātai Ngā Māngai Māori Board (review board to be contacted at nmm@matai.org.nz). This protocol ensures adherence to ethical standards, respects community involvement, and upholds data sovereignty principles.

A T1-weighted sequence was acquired to obtain a description of the cerebral anatomy from which the pial surface was extracted. This was accomplished by using a standard image segmentation procedure that rendered the cortical surface [42], composed of:

- the inner cortical surface: that is the interface between the gray matter and white matter, which is denoted by $\Gamma_{\text{GW}}$;
- the outer cortical surface, also known as the pial surface: that is the interface between the gray matter and the cerebrospinal fluid space, more specifically the *pia mater*, which is denoted by $\Gamma_{\text{pial}}$.

Although the proposed methodology can be applied to the entire brain, the present proof-of-concept study focuses on the vascularization and blood flow simulation in the pial circulation of the left hemisphere.

**Fig 1:**
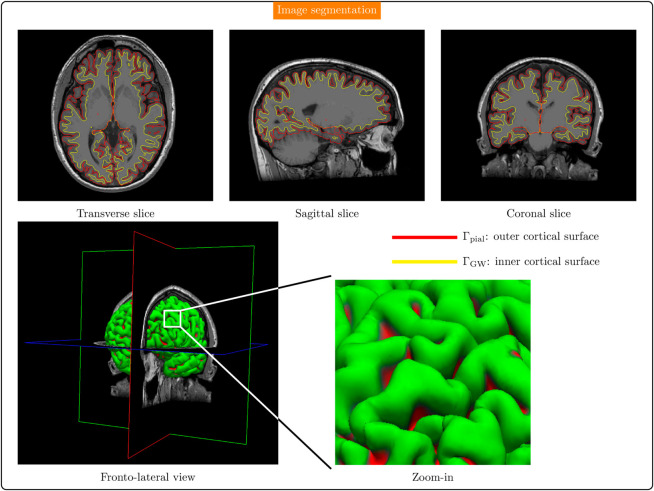
Segmentation of the magnetic resonance image dataset featuring the inner (yellow) and outer (red) cortical surfaces for three orthogonal planes. Sulci and gyri can be appreciated in the zoomed-in area performed over the fronto-lateral view.

Fig 1 shows the image segmentation results, providing the definition of the outer cortical surface $\Gamma_{\text{pial}}$, that is the pial surface, and of the inner cortical surface $\Gamma_{\text{GW}}$.

### Initial cerebral network

**Fig 2:**
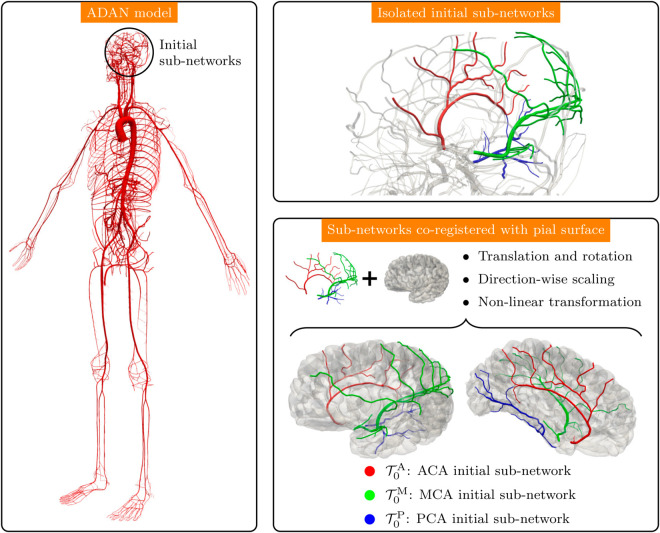
Preparation of the initial vascular network. Left: ADAN model. Right top: Selection of the initial vascular sub-networks $T_{0}^{A}$, $T_{0}^{M}$ and $T_{0}^{P}$ corresponding to the ACA, MCA and PCA territories, respectively. Right bottom: Non-linear co-registration of initial sub-networks on top of the pial surface.

We exploited the ADAN model (see Section ADAN model, and Fig 2, left panel) as an initial geometry from which the vascularization of the pial surface is to be extended. First, we isolated the three vascular sub-networks corresponding to the anterior cerebral artery (ACA), the middle cerebral artery (MCA), and the posterior cerebral artery (PCA), as shown in Fig 2, right top panel. To avoid closed-loop connections, the corpus callosum connection between ACA and PCA territories was disconnected. These initial sub-networks were registered on top of the pial surface generated in Section Patient-specific cerebrum through the following procedure (for more details, refer to [43]):

1. apply a translation to the ADAN model based on the displacement from the centroid of its Circle of Willis to the centroid of the Circle of Willis defined by the image-based geometry of the patient’s vessel centerlines;
2. apply a rotation consisting of the Rodrigues’ rotation formula to align the normals of the planes that better fit the patient CoW centerlines and the ADAN model CoW;
3. apply a scaling factor in each independent orthogonal direction. This factor is defined on the ratio between the standard deviation of the coordinates of the major points in the patient centerlines and the ADAN model centerlines;
4. apply a non-linear deformation mapping to accommodate the vessels over the pial surface. The coordinates of the model were projected onto the median surface between the inner and outer surfaces that define the pial surface (individual vertices were snapped to the face nearest point using Blender 3.4).

The result of the compounded co-registration procedure is shown in Fig 2, right bottom panel. These initial sub-networks are denoted by $T_{0}^{A}$, $T_{0}^{M}$ and $T_{0}^{P}$, for the ACA, MCA and PCA territories, respectively.

### Automatic vascularization

#### Pial space and vascular territories.

**Fig 3:**
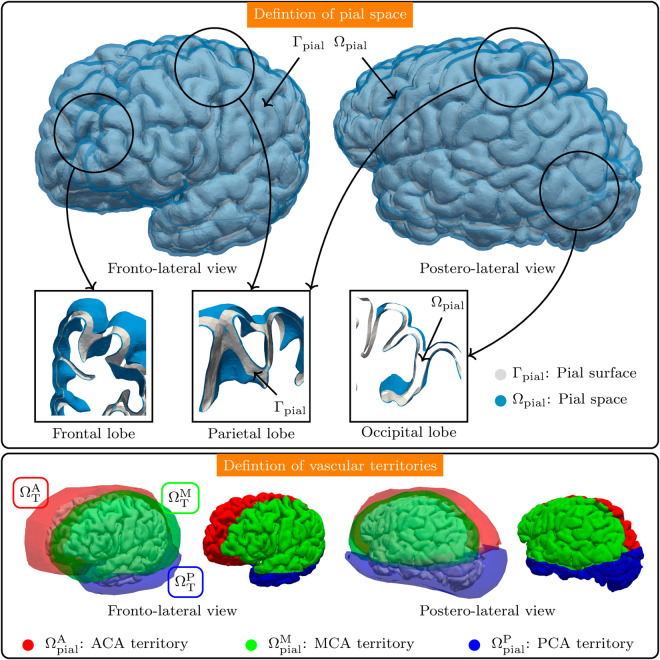
Pial space and vascular territories. Top: Generation of the pial space $\Omega_{\text{pial}}$ (in blue, with transparency) by extrusion of the pial surface $\Gamma_{\text{pial}}$ (in gray), and details of the pial space in different locations. Bottom: Definition of the ACA (red), MCA (green), and PCA (blue) vascular territories, by Boolean operations with the reference territorial solids $\Omega_{T}^{A}$, $\Omega_{T}^{M}$ and $\Omega_{T}^{P}$. These vascular spaces are denoted by $\Omega_{\text{pial}}^{A}$, $\Omega_{\text{pial}}^{M}$ and $\Omega_{\text{pial}}^{P}$, respectively. The corresponding pial surfaces are denoted by $\Gamma_{\text{pial}}^{A}$, $\Gamma_{\text{pial}}^{M}$, and $\Gamma_{\text{pial}}^{P}$.

The pial space is defined as the space where the pial vascular network is to be generated. This pial space was constructed by extruding the pial surface $\Gamma_{\text{pial}}$ in the direction defined by the local unit normal vector. The thickness of the pial space is 2 mm, and the extrusion was set 75% in the outward direction, and 25% in the inward direction, as shown in Fig 3, top panel. Thus, the pial space $\Omega_{\text{pial}}$ is defined as

$$\Omega_{\text{pial}}=\{x\in R^{3};x=x_{\text{pial}}+\xi n_{\text{pial}},\;x_{\text{pial}}\in \Gamma_{\text{pial}},\;\xi \in [-0.5,1.5]\},$$ (1)

where $n_{\text{pial}}(x_{\text{pial}})$ is the the unit normal vector to $\Gamma_{\text{pial}}$. The boundary $\partial \Omega_{\text{pial}}$ of the pial space was remeshed to remove geometric inconsistencies such as overlapping faces, and incorrectly oriented normals resulting of the extrusion of sharp edges.

The next step consisted in defining the major vascular territories corresponding to the ACA, MCA and PCA sub-networks. Following [44], we defined the territorial solids $\Omega_{T}^{A}$, $\Omega_{T}^{M}$, and $\Omega_{T}^{P}$, from where, we obtained three major pial territories through Boolean operations, by doing $\Omega_{\text{pial}}^{X}=\Omega_{T}^{X}\cap \Omega_{\text{pial}}$, X∈{A,M,P}, as shown in Fig 3, bottom panel. Similarly, we defined the corresponding ACA, MCA and PCA pial surfaces as $\Gamma_{\text{pial}}^{X}=\Omega_{T}^{X}\cap \Gamma_{\text{pial}}$, X∈{A,M,P}. We allowed a small overlap between adjacent territories in order to guarantee vascular redundancy in the furthest regions of the brain with respect to the corresponding inlet locations. Out of the total volume, 15% corresponds to overlapping domains. The total pial surface area resulted $|\Gamma_{\text{pial}}|=850{\text{cm}}^{2}$, and the total pial space volume was $|\Omega_{\text{pial}}|=170.2{\text{cm}}^{3}$, without considering the territorial overlapping. After the territorial definition, and taking into account the small overlap allowed, the surface areas and the corresponding enclosed volumes for the different territories were:

- $|\Gamma_{\text{pial}}^{A}|=315{\text{cm}}^{2}$, $|\Omega_{\text{pial}}^{A}|=63.6{\text{cm}}^{3}$;
- $|\Gamma_{\text{pial}}^{M}|=430{\text{cm}}^{2}$, $|\Omega_{\text{pial}}^{M}|=86.2{\text{cm}}^{3}$;
- $|\Gamma_{\text{pial}}^{P}|=230{\text{cm}}^{2}$, $|\Omega_{\text{pial}}^{P}|=46.8{\text{cm}}^{3}$.

Note that due to the overlap, we have $|\Gamma_{\text{pial}}^{A}|+|\Gamma_{\text{pial}}^{M}|+|\Gamma_{\text{pial}}^{P}|=975{\text{cm}}^{2}$, and $|\Omega_{\text{pial}}^{A}|+|\Omega_{\text{pial}}^{M}|+|\Omega_{\text{pial}}^{P}|=196.6{\text{cm}}^{3}$.

#### Space-filling vascular generation.

The Constrained Constructive Optimization algorithm has been extensively used to create anatomically consistent vascular networks in a variety of situations [36,37,45]. The CCO algorithm is based on a compartmental representation of a vascular network where mass and momentum balance laws are guaranteed, and where each vessel features a resistance based on the Poiseuille equation. The method relies on the optimization of a cost function to drive the sequential generation of vascular connections from randomly sorted locations in 3D space inside the geometrical boundary of the perfusion domain, so that each newly created connection satisfies a series of anatomical rules, such as a power-law relating vessel radii at junctions [46], bifurcation symmetry rules, and branching angle constraints.

We used the DCCO method proposed previously [39], which combines multiple stages with specific pre-defined vessel functions and additional constraints acting upon groups of vessels seeking to add versatility and enhance the overall modeling capabilities of the original CCO approach. However, due to the sequential nature of the CCO approach, its utilization for the construction of massive vascular networks is severely limited (computational cost scales with $N_{V}^{3}$, $N_{V}$: number of vessels). In this regard, [38] proposed a parallel and distributed version of the DCCO, called PDCCO, by enabling the vascularization process to be executed within a distributed computing paradigm. The PDCCO approach requires two inputs: (i) a baseline network, sequentially generated through the DCCO, which spans over the entire domain to be vascularized, and (ii) a decomposition of such a domain into disjoint sub-domains. The generation is concurrently executed in the different sub-domains, initiating from the baseline network, and allowing vessels to be added only within each specific sub-domain. After the concurrent generation is finished, all the networks are merged into the definitive full network ensuring that the anatomical constraints and balance laws are all verified in the full network. For more details, the reader is referred to [38].

**Fig 4:**
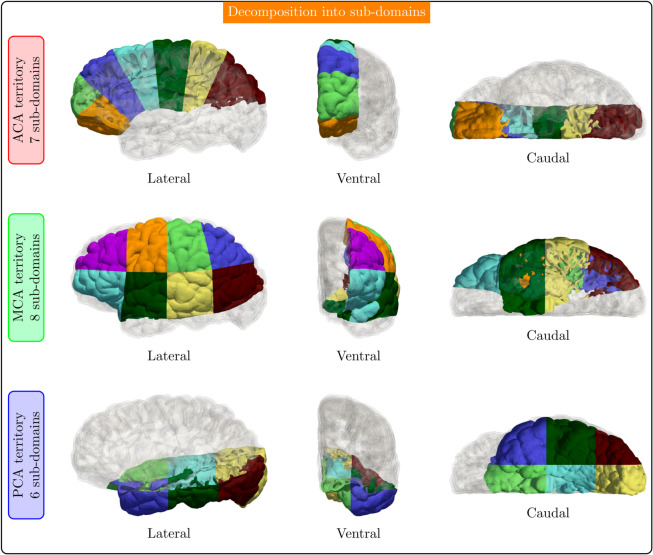
Decomposition into sub-domains for the ACA (top row), MCA (middle row) and PCA (bottom row) territories. Each color represents a sub-domain. The projections from the lateral, ventral, and caudal directions are shown in each case.

In this work, we employed the PDCCO algorithm, applying it to each one of the three sub-networks corresponding to the ACA, MCA and PCA territories. Thus, in a nutshell, we took each one of these three territories, first generated a baseline sub-network, created a decomposition of each vascular territory into sub-domains and performed the vascularization/merging as described in [38] (see PDCCO parameter setting in the next section). Fig 4 illustrates the partitioning into sub-domains for the ACA, MCA and PCA territories.

The PDCCO method relies on a 0D purely resistive model for the blood flow in pipes, in which mass conservation is ensured at each vessel junction, and in each vessel Poiseuille flow is assumed to hold. Noting that we aim to generate massive vascular networks wherein vessel diameters will reach values in the order of tens of microns, it is important to account for the rheology of blood. Hence, we used the viscosity model proposed by Pries et al. [47] in all the vascularization stages.

#### Vascularization stages.

To describe the vascularization strategy, we employ the nomenclature and symbols defined by Talou et al. [39]. For a detailed account of the parameters and overall procedure, see that bibliographic reference. Here we do not reproduce the equations and overall methodology, but we provide a brief description of the stage architecture and all the parameters involved in the setting of the vascularization algorithm. First, we considered the following multi-staged procedure:

$S_{1}$: (first stage) grows out of the initial sub-networks by covering the pial space using a sprouting cost functional;
$S_{2}$: (second stage) extends the networks created in the first stage by using a volumetric cost functional;
$S_{3}$:(third stage) performs a massive parallel vascularization from networks created in the second stage by using a volumetric cost functional and the domain decomposition explained in Section Space-filling vascular generation.

Next, the model parameters were defined to characterize the vascular networks in each of these stages. These parameters are the following:

- *Q*_*i*_: mean flow rate prescribed at inflow in ml/min (taken from [33] as the time average inlet flow for each major cerebral territory after the circle of Willis);
- *N*_*T*_: number of terminal vessels in the network targeted for each stage;
- $P_{\text{geo}}$: geometric parameters:
  – *γ*: power-law parameter to relate parent and children vessels,
  – *δ*: radius symmetry ratio at bifurcations,
  – $\theta_{\text{min}}$: minimum branching angle between parent and children vessels,
  – $\phi_{\text{min}}$: minimum out-of-plane opening angle;
- $P_{\text{opt}}$: optimization parameters:
  – ν: perfusion area factor,
  – *f*_*r*_: region reduction factor after 2 000 unsuccessful candidates,
  – *f*_*n*_: neighborhood search factor,
  – Δv: number of points used to discretize edges of the triangle generated when a new connection candidate is evaluated, which defines the discrete mesh where the optimal connection is established;
- Distribution vessel type: the vessel bifurcates only if it is located entirely within the domain.

Table 1 presents the parameters employed in the vascularization stages for the setting of the PDCCO algorithm. The sprouting cost functional employed in stage $S_{1}$ was configured with $C_{v}=1\;\cdot \;10^{4}$, *C*_*p*_ = 0.5, *C*_*d*_ = 1. Then, a volumetric cost functional was used for the two subsequent stages.

**Table 1:** Parameters to configure the multi-staged vascularization using the PDCCO algorithm. For the definition of parameters, see Section Vascularization stages.

Stage	Domain	$Q_{inlet}$ [ml/min]	$N_{T}$	$P_{geo}$ $\left(\gamma ,\delta ,\theta_{min},\phi_{min}\right)$	$P_{opt}$ $(\nu ,f_{r},f_{n},\Delta v)$	Input/output network	Cost function	Vessel type
$S_{1}$	$\Omega_{\text{pial}}^{A}$	70	1300	$\left(3,0.4,20^{\circ },0^{\circ }\right)$	(0.01,0.9,100,7)	$T_{0}^{A}$/$T_{1}^{A}$	$F_{\text{sprout}}$	Distribution
	$\Omega_{\text{pial}}^{M}$	125	1700	$\left(3,0.4,20^{\circ },0^{\circ }\right)$	(0.01,0.9,100,7)	$T_{0}^{M}$/$T_{1}^{M}$	$F_{\text{sprout}}$	Distribution
	$\Omega_{\text{pial}}^{P}$	65	1000	$\left(3,0.4,20^{\circ },0^{\circ }\right)$	(0.01,0.9,100,7)	$T_{0}^{P}$/$T_{1}^{P}$	$F_{\text{sprout}}$	Distribution
$S_{2}$	$\Omega_{\text{pial}}^{A}$	70	6500	$\left(3,0,20^{\circ },0^{\circ }\right)$	(0.01,0.9,100,7)	$T_{1}^{A}$/$T_{2}^{A}$	$F_{\text{vol}}$	Distribution
	$\Omega_{\text{pial}}^{M}$	125	8500	$\left(3,0,20^{\circ },0^{\circ }\right)$	(0.01,0.9,100,7)	$T_{1}^{M}$/$T_{2}^{M}$	$F_{\text{vol}}$	Distribution
	$\Omega_{\text{pial}}^{P}$	65	5000	$\left(3,0,20^{\circ },0^{\circ }\right)$	(0.01,0.9,100,7)	$T_{1}^{P}$/$T_{2}^{P}$	$F_{\text{vol}}$	Distribution
$S_{3}$	$\Omega_{\text{pial}}^{A}$	70	37803	$\left(3,0,20^{\circ },0^{\circ }\right)$	(0.01,0.9,100,7)	$T_{2}^{A}$/$T_{3}^{A}$	$F_{\text{vol}}$	Distribution
	$\Omega_{\text{pial}}^{M}$	125	51595	$\left(3,0,20^{\circ },0^{\circ }\right)$	(0.01,0.9,100,7)	$T_{2}^{M}$/$T_{3}^{M}$	$F_{\text{vol}}$	Distribution
	$\Omega_{\text{pial}}^{P}$	65	27579	$\left(3,0,20^{\circ },0^{\circ }\right)$	(0.01,0.9,100,7)	$T_{2}^{P}$/$T_{3}^{P}$	$F_{\text{vol}}$	Distribution

The set of terminal vessels in network $T_{3}$ is denoted by $ter(T_{3})$, and each sub-network in the different territories is denoted as $ter(T_{3}^{X})$, where X∈{A,M,P}. The final number of terminal vessels *N*_*T*_ after completion of the final stage $S_{3}$, in $T_{3}$, that is, the cardinality of the set $ter(T_{3})$, was chosen to reach a vascular density of $1.2{\text{terminals/mm}}^{2}$ (for the surface area of each territory, refer to Section Pial space and vascular territories). In this context, each terminal vessel in the set $ter(T_{3})$ would correspond to a descending arteriole that penetrates the gray matter and provides blood to a certain columnar domain [48]. This terminal vessel density is in agreement with Schmid et al. [40], where the authors reported a density of one descending arteriole per square millimeter.

#### Inter-territorial pressure distribution equalization.

In this section we describe an important step to deliver consistent results across the different territories. Consistency here must be understood in the sense of scaling network diameter values for each territory such that, for the flow supplied to each of them, the resulting pressure distributions in all networks are as similar as possible. We term this procedure as pressure distribution equalization.

Note that the inflow boundary conditions defined in Table 1 and the initial sub-networks $T_{0}^{X}$, X∈{A,M,P} were taken from Blanco et al. [33]. The vessel diameters in these sub-networks were defined in the same reference according to typical values encountered in the literature. However, the matching between inlet vessel diameter and the flow rate supplied to each territory was not specifically calibrated at the time of these publications. In fact, a quick assessment in the light of the Murray’s Law, that is considering the relation between the flow rate and the cube of the inlet vessel radius ($Q=\xi r^{3}$, *r*: vessel radius, ξ: proportionality constant, see [46,49]) yields the data reported in Table 2. Clearly, the different values of ξ obtained for the different territories indicate that the flow rate/network diameter relation is not the same across the territories, which can be explained by the fact that the flow data does not correspond to the specific patient anatomic geometry and therefore vessel diameters. This was manifested in the vascularization process, where the networks $T_{3}^{A}$, $T_{3}^{M}$, and $T_{3}^{P}$ rendered pressure distributions that were not equalized. Observe that these pressure distributions are obtained under the steady-state assumption that governs the CCO vascularization process. To mitigate this lack of consistency across the pairs $\left(T_{0}^{X},Q_{\text{inlet}}^{X}\right)$, X∈{A,M,P}, from the ADAN model, we found an optimal scaling factor *f*_*S*_ that affects all vessel diameters for each network such that the pressure distributions rendered by the PDCCO algorithm (i.e. from the steady state Poiseuille model) were equalized. To perform this equalization process we computed an optimal scaling factor over the networks $T_{3}^{A}$ and $T_{3}^{P}$ such that the quadratic error between the corresponding pressure distributions and that one delivered by the network $T_{3}^{M}$, was minimized. In approximating these distributions we shifted the pressure value at the inlet vessel corresponding to the circle of Willis to 80 mmHg, and we took 1000 bins uniformly distributed in the range [0,80] mmHg. Table 2 also shows the actual inlet radius that results after the equalization procedure, and the same proportionality factor after the equalization.

**Table 2:** Data and parameters regarding inter-territorial pressure distribution equalization. $Q_{\text{inlet}}$: flow rate at the inlet, $r_{\text{inlet}}^{\mathrm{ADAN}}$: inlet vessel radius given in ADAN model, $\xi^{\mathrm{ADAN}}$: proportionality constant in Murray’s law for ADAN model, *f*_*S*_ scaling factor for vessel diameters, $r_{\text{inlet}}$: inlet vessel radius after equalization, and ξ: proportionality constant in Murray’s law for the scaled network after equalization.

Network	Q_inlet_ [ml/min]	$r_{inlet}^{ADAN}$ [μm]	*ξ*^ADAN^ [1/s]	*f*_s_	*r*_inlet_ [μm]	*ξ* [1/s]
$T_{3}^{A}$	70	965	1 298	0.895	864	1 809
$T_{3}^{M}$	125	1 041	1 847	1.000	1 041	1 847
$T_{3}^{P}$	65	869	1 651	0.915	795	2 156

### ADAN model

We made use of the anatomically detailed arterial network (ADAN) model developed by Blanco et al. [33] as a framework:

- to provide an initial vascular architecture of the cerebral vessels to be expanded, as explained in Section Initial cerebral network; and
- to be coupled with the automatically-generated vascular networks of the pial circulation to carry out one-dimensional blood flow simulations.

Notably, the use of the ADAN model adds negligible computational burden to the simulations (see Section Numerical method). However, the most significant reason for coupling the ADAN model to the inlets of the CCO networks was to handle forward-backward traveling waves between the cerebral network and the rest of the arterial system consistently. This is crucial in avoiding the introduction of boundary artifacts in microcirculatory hemodynamics due to poorly defined boundary conditions.

The ADAN model comprises 2142 arteries, including 1598 arterial segments with a well-acknowledged name and 544 perforator arteries. This model has been used to investigate complex hemodynamic phenomena such as subclavian steal syndrome [50], and, in line with the present study, cerebral blood pressure gradients [32].

**Fig 5:**
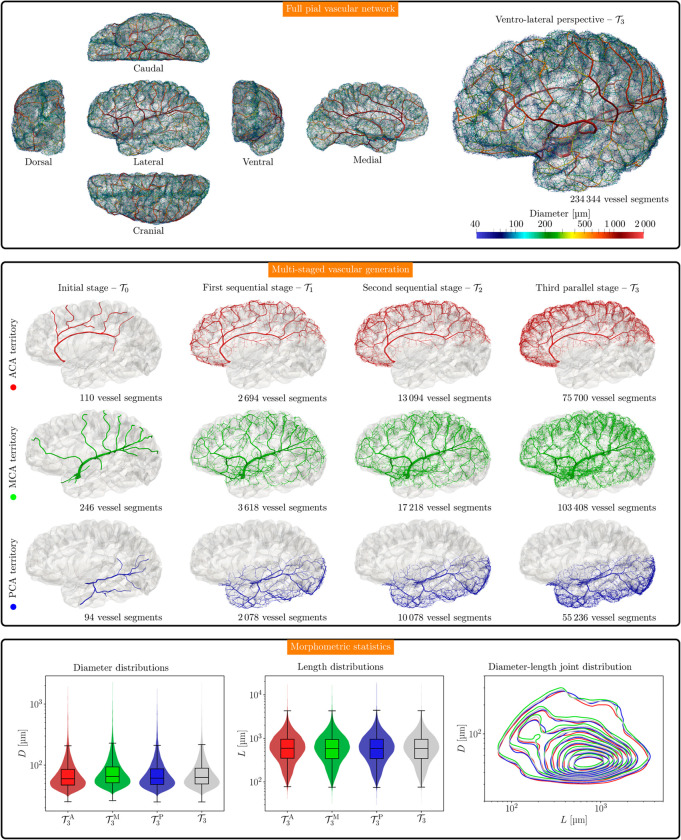
Pial vascular network built with the proposed multi-stage approach. Top panel: Orthogonal projections on transverse, coronal and sagittal planes, and ventro-lateral perspective, with color-code given by vessel diameter in μm. Mid panel: Multi-staged vascularization of the pial space. From left to right: initial sub-networks from the ADAN model (totaling 450 vessel segments), first and second sequential vascularization stages with different cost functions (totaling 8 390 and 40 390 vessel segments, respectively), and third vascularization stage after merging the parallelized sub-domain networks (totaling 234 344 vessel segments). Bottom panel: Distribution of vessel diameters *D*, vessel lengths *L*, and their joint kernel density estimate distribution in the pial networks for each of the three territories, $T_{3}^{A}$ (red), $T_{3}^{M}$ (green), and $T_{3}^{P}$ (blue), and also for the entire pial network $T_{3}$ (gray). All distributions are in logarithmic scale.

The three vascular territories generated in Section Automatic vascularization were coupled to their corresponding locations within the ADAN model, resulting in a natural expansion of the original ADAN model, now accounting for the highly detailed description of the pial circulation. The vascular network featured in the top panel of Fig 5 replaces the original cerebral vascular networks in the ADAN model shown in the right panels of Fig 2. Mathematically, ADAN and CCO networks are coupled following mass and energy conservation at junctions, as described in Section Blood flow model.

Importantly, no perforator arteries are present in the cerebral vascular networks of the ADAN model that are expanded by the CCO vascularization process. The original ADAN model contains small perforator-like vessels around the circle of Willis. These vessels supply small structures at the base of the brain (e.g., lenticulostriate, thalamotuberal, hypothalamic arteries, among others), and remained unaffected after coupling the CCO networks to the ADAN model.

### Blood flow model

Blood flow in the vascular network is described following the 1D theory of incompressible fluid flow in deformable vessels. The 1D mass and momentum conservation laws (see derivation in [41]) describe the pressure *P*, flow rate *Q*, and lumen area *A* as a function of time *t* and the longitudinal coordinate *x*, as follows

$$\frac{\partial A}{\partial t}+\frac{\partial Q}{\partial x}=0,$$ (2)

$$\frac{\partial Q}{\partial t}+\frac{\partial }{\partial x}(\frac{Q^{2}}{A})=-\frac{A}{\rho }\frac{\partial P}{\partial x}-\frac{8\pi \mu }{\rho A}Q,$$ (3)

where *ρ* and *μ* are the blood density and viscosity, respectively. The vessel wall is considered to be a nonlinear viscoelastic structure, and under the thin-wall assumption, mechanical equilibrium determines the following relation between pressure and lumen area

$$P=P_{0}+\frac{\pi r_{0}h}{A}(E_{e}\varepsilon +E_{c}\epsilon_{r}\ln({\text{e}}^{\chi }+1)+\frac{K_{m}}{2\sqrt{AA_{0}}}\frac{\partial A}{\partial t}),$$ (4)

$$\varepsilon =\sqrt{\frac{A}{A_{0}}}-1,\;\chi =\frac{\varepsilon -\epsilon_{0}}{\epsilon_{r}},$$ (5)

where $A_{0}=\pi r_{0}^{2}$ is the reference lumen area at the reference pressure *P*_0_, *h* is the wall thickness, *E*_*e*_ and *E*_*c*_ are the effective elastic moduli of the elastin and collagen fibers, $\epsilon_{0}$ and $\epsilon_{r}$ are mean and standard deviation of the fiber recruitment normal distribution function, and *K*_*m*_ is the viscoelastic coefficient.

At junctions, with *N* converging vessels, coupling conditions stand for conservation of mass and total energy, that is

$$\sum_{i=1}^{N}Q_{i}=0,$$ (6)

$$P_{1}+\frac{\rho }{2}(\frac{Q_{1}}{A_{1}}{)}^{2}=P_{i}+\frac{\rho }{2}(\frac{Q_{i}}{A_{i}}{)}^{2}\;i=2,\ldots ,N.$$ (7)

Peripheral boundary conditions are modeled using 3-element Windkessel elements, which provide a closure equation at the end of each terminal vessel that relates the outlet flow rate *Q*_*o*_ to the outlet pressure *P*_*o*_ as follows

$$R_{1}R_{2}C\frac{dQ_{o}}{dt}=R_{2}C\frac{d}{dt}(P_{o}-P_{t})+(P_{o}-P_{t})-(R_{1}+R_{2})Q_{o},$$ (8)

where *R*_1_, *R*_2_, and *C* are the parameters that represent the peripheral resistances and compliance downstream to each terminal vessel, and *P*_*t*_ is the reference pressure.

In the 1D simulations, we used the same non-Newtonian constitutive model for the blood employed in the PDCCO vascularization process [47], where the viscosity is a function of the vessel radius $\mu_{\text{NN}}(r_{0})$.

### Numerical method

The blood flow Eqs (2), (3) and (4) constitute an advection-diffusion-reaction system of partial differential equations (PDEs). This system is recast in hyperbolic form following the strategy presented in [51,52] to obtain a system of first order PDEs. The hyperbolized system is discretized in time and space using an explicit, local time-stepping, second order finite volume scheme, as per [53]. This approach ensures high-order accuracy also at junctions [54]. The method also considers consistent coupling conditions at junctions of viscoelastic vessels, as discussed in [55].

The maximum value of the local time step was $\Delta t_{max}=1\;ms$, and the time step in each vessel was determined such that time synchronization is guaranteed at all junctions, while the following Courant-Friedrichs-Lewy stability condition was maintained *CFL*<0.9. Concerning the spatial discretization, the characteristic cell length was set to $\Delta x_{c}=1\;cm$, and smaller vessels were discretized with a single computational cell. The ordinary differential equation of the Windkessel model (8) was discretized with an explicit Euler method, and its coupling to 1D terminal vessels is described in [53].

All simulations shown in this work were performed in the Santos Dumont high performance facility available at the LNCC (MCTI, Brazil), using 5 computational nodes, each equipped with 2x Intel Xeon Cascade Lake Gold 6252, for a total of 48 cores per node, which amounts to 240 cores allocated for each simulation. To ensure periodic state, we simulated 10 cardiac cycles, and used the results of the last cycle for the analysis. Total simulation running time was approximately 80h. This large computational burden is primarily driven by the explicit numerical method used to discretize the blood flow model in the case of viscoleastic vessel walls. For more details about the computational cost in 1D simulations using the present numerical method, the reader is referred to [53].

It is worthwhile to note that the overall computational burden is attributed to the large number of vessels present in the CCO networks. Moreover, since these vessels are the smallest ones, the time-step selection to meet the *CFL* stability condition depends exclusively on the length of these vessels. This, in addition to the use of a local time-stepping technique, reduces the number of computations that involve the vessels of the ADAN network. The number of vessels in the coupled ADAN-CCO model is 238385, with 1.7% being from the ADAN model (4041 vessels) and 98.3% from the CCO model (234344 vessels). The number of computational cells is 245006, which shows that most of the cells pertain to the CCO network. Regarding the time step, the average time step in the ADAN-CCO coupled model results $\Delta t_{\text{avg}}=5.7\;\cdot \;10^{-6}\;s$, while the minimum and maximum values are $\Delta t_{\text{min}}=1.22\;\cdot \;10^{-7}\;s$ and $\Delta t_{\text{min}}=5.0\;\cdot \;10^{-4}\;s$, respectively. As a consequence, the use of the ADAN model incurs negligible additional cost in the computations.

### Parameter setting

Simulations were performed employing the ADAN model coupled to the CCO networks of the ACA, MCA and PCA territories. We considered two physiological scenarios of interest, the normotensive condition, and the hypertensive condition. For the normotensive setting, all the parameters for the ADAN-CCO coupled model were calibrated according to the criteria presented in [56]. Specifically, we have

- Terminal parameters ($R_{1},R_{2},C$) in the ADAN model were calibrated following the procedure described in [57] to ensure a correct blood flow distribution among the different organs and vascular territories.
- Terminal parameters ($R_{1},R_{2}$) in the CCO model are purely resistive and were calibrated such that they guarantee a uniform flow rate across all network outlets, as done in [32].
- Lumen area (*A*_0_) in the ADAN-CCO coupled model was retrieved from the specialized literature, as described in [33].
- Arterial wall thickness (*h*) in the ADAN-CCO coupled model was computed following the relation reported in [33], obtained by fitting data from [58].
- Material parameters ($E_{e},E_{c},K_{m},\epsilon_{r},\epsilon_{0}$) in the ADAN-CCO coupled model were calibrated following the criteria used in [56].

For the hypertensive setting, the parameters were modified following a similar strategy to that proposed by Blanco et al. [32], that is, applying a multiplication factor *f*_*H*_ as described in Table 3. Peripheral resistances in the CCO networks were scaled differently than the rest of the ADAN network because part of the resistance is found in the pial network itself. These factors were such that both hemispheres receive the same average blood flow.

**Table 3:** Multiplication factors for the modification of parameters in the hypertensive scenario.

Model	ADAN	ADAN	CCO	ADAN+CCO		
Parameter	$R_{1},R_{2}$	*C*	$R_{1},R_{2}$	*E*_*e*_	*h*	*A*_0_
*f*_*H*_	1.40	0.85	1.47	2.0	1.4	0.81

In addition to the viscoelastic scenario used as the reference solution, we also considered alternative modeling scenarios to assess the impact of modeling hypotheses. Particularly, we considered removing viscoelasticity from the model, that is setting *K*_*m*_ = 0 in (4), and assumed Newtonian blood behavior, which implies $\mu_{\text{N}}=0.032\;P$. This assumption for the Newtonian model is consistent with the asymptotic value of the viscosity delivered by the non-Newtonian model $\mu_{\text{NN}}$ for large values of vessel radius *r*_0_, corresponding to a hematocrit of 0.45 (see also [47]).

### Statistical analysis

Scenarios were analyzed not only by investigating the hemodynamics over time, that is pressure *P*(*t*), and flow rate *Q*(*t*), but also in terms of maximum, minimum and average values along the cardiac cycle. The descriptive statistics were computed for the population of vessels in the vascular network. The maximum (systolic), minimum (diastolic) and mean values, of pressure and flow rate (Z∈{P,Q}), are defined as

$$Z_{\text{s}}=\max_{t\in [0,T]}Z(t),$$ (9)

$$Z_{\text{d}}=\min_{t\in [0,T]}Z(t),$$ (10)

$$Z_{\text{m}}=\frac{1}{T}\int_{0}^{T}Z(t)\;dt.$$ (11)

Also, for selected paths, whose longitudinal coordinate is denoted by x∈[0,L], *x* = 0 being the root of the corresponding territory, and *x* = *L* the final terminal point of the path (with total length *L*), the pressure is reported as a contour plot in the (*t*,*x*) space, that is *P*(*t*,*x*), so that the wave propagation in the spatio-temporal domain can be visualized. Maximum, minimum and average pressure values, i.e. $P_{\text{s}}(x)$, $P_{\text{d}}(x)$, and $P_{\text{m}}(x)$, are also reported along the paths, as functions of the distance traveled through the path.

In addition, we defined the pulsatility index PI from the flow rate waveform as follows

$$\text{PI}=\frac{Q_{\text{s}}-Q_{\text{d}}}{Q_{\text{m}}},$$ (12)

where $Q_{\text{s}}$, $Q_{\text{d}}$, and $Q_{\text{m}}$ are the systolic, diastolic and mean flow respectively, and the pulse pressure PP as

$$\text{PP}=P_{\text{s}}-P_{\text{d}}.$$ (13)

From these, we defined the damping factors DF for the pulsatility index and for the pulse pressure as follows

$${\mathrm{DF}}_{Y}=\frac{\max_{T_{3}}Y}{Y}\;Y\in \{\text{PI},\text{PP}\},$$ (14)

where $\max_{T_{3}}Y$ is the maximum value reached by index Y∈{PI,PP} in the network $T_{3}$. Observe that ${\mathrm{DF}}_{\text{PI}}$ and ${\mathrm{DF}}_{\text{PP}}$ are quantities that vary along $T_{3}$, which which can help to unveil a variable damping phenomenon of the energy through the different regions of the brain. Expression (14) is a generalization of the methodology to compute the damping factors used in the clinic, where the numerator is a reference value for quantity Y, which is typically measured at a proximal location, and the denominator is the same quantity measured at a distal location. For the sake of generality, in (14) we chose $\max_{T_{3}}Y$ as the reference value.

## Results

### Vascular morphometry

In this section we describe the morphometric features of the pial vascular networks $T_{3}^{X}$, X∈{A,M,P} in terms of vessel diameters and lengths. We also report the results for the union of these three networks, that is $T_{3}=T_{3}^{A}\cup T_{3}^{M}\cup T_{3}^{P}$.

The pial vascular network $T_{3}$ resulting from the proposed multi-stage approach is shown in Fig 5, top panel. In turn, the corresponding vascular networks, $T_{k}^{A}$, $T_{k}^{M}$, and $T_{k}^{P}$, k∈{0,1,2,3}, through the consecutive stages can be seen in Fig 5, mid panel. Considering the final sub-networks $T_{3}^{X}$, X∈{A,M,P}, and the entire network $T_{3}$, Fig 5, bottom panel, presents, in logarithmic scale, the distributions of vessel segment diameters (left), the distributions of the vessel lengths (mid), and their joint kernel density estimate (right). Mean, median, standard deviation and lower and upper quartiles for the vessel diameters and lengths in the different territories, and for the entire network, are reported in the top half part of Table 4. The cumulative vessel length, the cumulative vascular volume, and the number of vessel segments in each network are reported in the same table. Recall that in the three territories we considered a density of 1.2 terminal segments per ${\text{mm}}^{2}$, so this feature is maintained along the three territories. The bottom half of Table 4 reports the statistics for the set of terminal vessel segments, denoted by ter(·) for network (·).

**Table 4:** Statistics for vessel diameter *D* and vessel length *L* in the pial vascular networks for the different territories and for the entire network. $\overset{―}{\left(\;\cdot \;\right)}$: mean value, $(\;\cdot \;{)}_{\sigma }$: standard deviation, $\tilde{\left(\;\cdot \;\right)}$: median value, $\left[(\;\cdot \;{)}_{Q_{1}},(\;\cdot \;{)}_{Q_{3}}\right]$: lower and upper quartiles. *L*_*T*_: cumulative vessel length, $V_{T}$: cumulative vascular volume, $N_{V}$: number of vessels.

Network	Vessel diameter [μm]		Vessel length [μm]		Cumulative		
	$\overset{―}{D}\;(D_{\sigma })$	$\tilde{D}\;[D_{Q_{1}},D_{Q_{3}}]$	$\overset{―}{L}\;(L_{\sigma })$	$\tilde{L}\;[L_{Q_{1}},L_{Q_{3}}]$	$L_{T}$ [mm]	$V_{T}$ $\left[{\text{mm}}^{3}\right]$	$N_{V}$
$T_{3}^{A}$	78.6 (67.9)	60.0 [47.2,85.3]	731 (567)	591 [349,953]	55 345.1	854.88	75 700
$T_{3}^{M}$	85.4 (70.6)	65.6 [51.4,93.5]	728 (584)	585 [342,941]	75 246.0	1 442.78	103 408
$T_{3}^{P}$	80.1 (65.9)	61.2 [48.3,87.1]	733 (595)	587 [343,953]	40 512.6	579.55	55 236
$T_{3}$	82.0 (68.7)	62.8 [49.3,89.3]	730 (581)	587 [345,948]	171 103.7	2 877.21	234 344
Set of terminal vessel segments ter(·)							
$ter(T_{3}^{A})$	49.3 (10.2)	47.5 [41.7, 55.1]	876 (570)	739 [492,1112]	–	–	37 803
$ter(T_{3}^{M})$	53.6 (11.1)	51.7 [45.3, 60.0]	859 (581)	720 [475,1088]	–	–	51 595
$ter(T_{3}^{P})$	50.4 (10.4)	48.6 [42.7, 56.2]	872 (600)	728 [483,1099]	–	–	27 579
$ter(T_{3})$	51.1 (10.8)	49.7 [43.4, 57.6]	868 (582)	728 [482,1099]	–	–	116 977

### Hemodynamics in the human cortex

Here we report a comprehensive characterization of the hemodynamics according to the pulsatile 1D blood flow model of the cortex vascular network $T_{3}$ for the normotensive and hypertensive conditions studied in this work.

**Fig 6:**
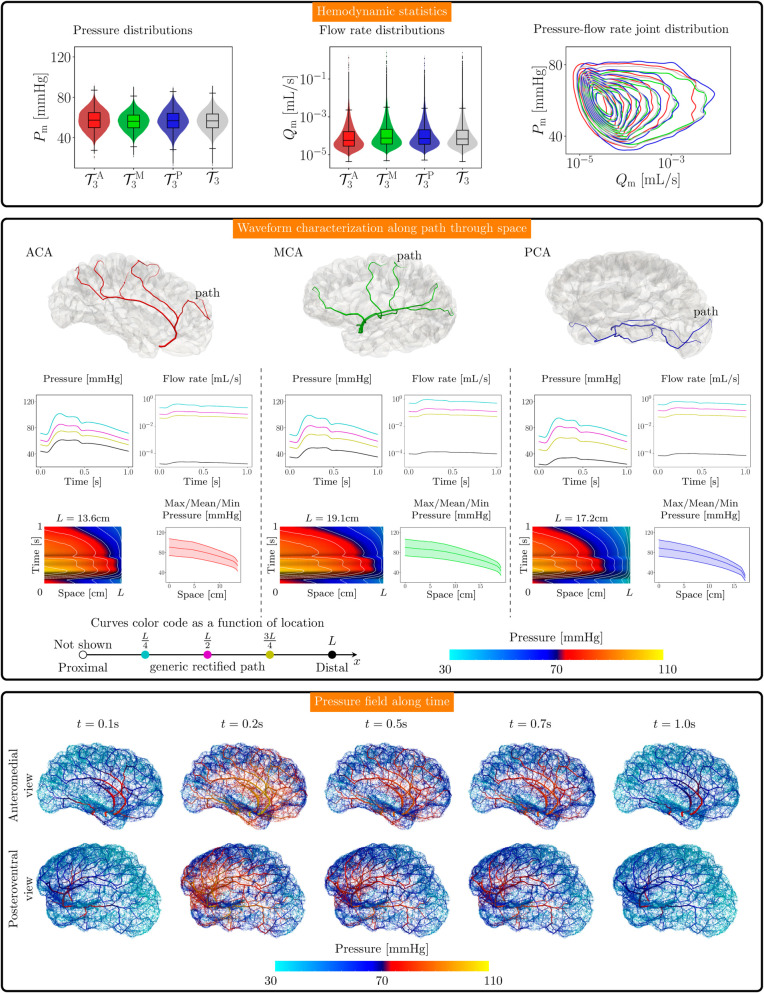
Hemodynamics in the cerebral cortex for the normotensive scenario. Flow rate is reported in logarithmic scale. Top panel: Distribution of mean blood pressure over time $P_{\text{m}}$, mean flow rate over time $Q_{\text{m}}$, and their joint kernel density estimate distribution in the pial networks for each of the three territories $T_{3}^{A}$ (red), $T_{3}^{M}$ (green), and $T_{3}^{P}$ (blue), and also for the entire pial network $T_{3}$ (gray). Mid panel: Pressure (top left inset) and flow rate (top right inset) waveforms along four evenly spaced locations placed in three arbitrarily chosen paths with length *L* (one per territory); pressure through space is characterized in the space-time domain (bottom left inset, isolines in white), and using the maximum, mean and minimum temporal values of the pressure waveforms along each path (bottom right inset) from the corresponding territory root to the terminal point at distance *L*. Bottom panel: Visualization of the blood pressure in each vessel along the entire cortex network for five different time-instants during the cardiac cycle.

For the normotensive case, mean (standard deviation), and median [lower, upper quartiles] values for the blood pressure and flow rate in the different territories, and for the entire network, are reported in Table 5. Fig 6 presents data for the normotensive scenario. Fig 6, top panel, presents the statistics for the blood pressure (left) and flow rate (mid), as well as their joint kernel density estimate distribution (right), from where we can see the skewed aspect of the flow rate distribution in the cortical network. Then, we arbitrarily selected three vascular paths, one per vascular territory, and characterized the hemodynamics along these paths. Fig 6, mid panel, features, for each path, the blood pressure (top left inset) and the flow rate (top right inset, in logarithmic scale) waveforms at four locations evenly spaced along each path. The shape of the pressure waveform as a space-time field is also displayed (bottom left inset), to characterize the variation of the pressure tracing as we move from the proximal location (root of the corresponding vascular network) to the terminal point at the proposed penetrating vessel location. The length of each path, denoted by *L*, is also reported in the figure. In addition, the systolic (maximum), mean, and diastolic (minimum) pressure values as a function of the longitudinal coordinate along each path are reported (bottom right inset). The pressure gradient was not significantly different for the different territories, and what differed was actually the length through which the gradient acts upon to define the pressure level, and the pressure drop increased in the last section of the paths. In turn, Fig 6, bottom panel, shows the blood pressure as a field in the entire vascular network $T_{3}$ for five selected time instants during the cardiac cycle, namely t∈{0.1,0.2,0.5,0.7,1.0}s. In these spanshots, we can see the highly non-homogeneous nature of the pressure field regardless of the instant along the cardiac cycle, which resulted in a persistent non-homogeneous cortical perfusion pressure.

**Table 5:** Descriptive statistics for temporal mean pressure $P_{\text{m}}$ and mean flow rate $Q_{\text{m}}$ in the pial vascular networks for the different territories and for the entire network in the normotensive scenario. $\overset{―}{\left(\;\cdot \;\right)}$: mean value, $(\;\cdot \;{)}_{\sigma }$: standard deviation, $\tilde{\left(\;\cdot \;\right)}$: median value, $\left[(\;\cdot \;{)}_{Q_{1}},(\;\cdot \;{)}_{Q_{3}}\right]$: lower and upper quartiles. $N_{V}$: number of vessels.

Network	Pressure [mmHg]		Flow rate $10^{-5}[ml/s]$		
	${\overset{―}{P}}_{m}\;(P_{m,\sigma })$	${\tilde{P}}_{m}\;[P_{m,Q_{1}},P_{m,Q_{3}}]$	${\overset{―}{Q}}_{m}\;(Q_{m,\sigma })$	${\tilde{Q}}_{m}\;[Q_{m,Q_{1}},Q_{m,Q_{3}}]$	$N_{V}$
$T_{3}^{A}$	57.3 (10.0)	57.4 [49.9,64.8]	139.8 (2 266)	5.7 [2.8,16.5]	75 700
$T_{3}^{M}$	56.1 (8.6)	56.2 [49.8,62.4]	201.0 (3 767)	7.6 [3.7,22.2]	103 408
$T_{3}^{P}$	56.8 (10.4)	56.8 [49.7,64.1]	137.6 (1 994)	7.1 [3.6,20.6]	55 236
$T_{3}$	56.7 (9.5)	56.7 [49.8,63.5]	166.3 (2 977)	6.9 [3.4,19.9]	234 344

The systolic, mean and diastolic pressure values for different vascular districts in the model are reported in Table 6 for both normotensive and hypertensive scenarios. Specifically, that table reports the systolic/mean/diastolic blood pressure values at the left brachial artery (BA), the proximal left internal carotid artery (l. ICA p.), the distal left internal carotid artery (l. ICA d.), and the corresponding median values for different arterial vessel subsets within the three networks $T_{3}^{A}$, $T_{3}^{M}$ and $T_{3}^{P}$, considering different vessel diameters, and different ranges of the vessel distance to the root of the territory. Considering the normotensive condition as the reference value, the systolic pressure overall increased approximately 62%, while the mean pressure increased 37% and the diastolic pressure was slightly reduced by 5%. From the same table it can be seen that while vessel diameter had a mild correlation with the pressure levels (the smaller the vessel, the lower the pressure), the main factor determining the pressure level in a vessel was the distance to the root of the territory. Vessels located at more distal locations feature a substantially reduced blood pressure, consistently among the three territories. Moreover, the PCA territory was the one that features the lowest (median) pressure levels, and this happened not only for small vessels (40μm<D≤60μm), but also for mid-sized arterioles (260μm<D≤280μm). For same-sized arterioles, it can also be seen that the pressure gradient between proximal and distal locations was larger in hypertension than the normotensive case.

**Table 6:** Comparison of the systolic $P_{\text{s}}$, mean $P_{\text{m}}$ and diastolic $P_{\text{d}}$ pressure values at different locations for the normotensive and hypertensive scenarios. BA: brachial artery, l. ICA: left internal carotid artery, p.: proximal, d.: distal, $\tilde{\left(\;\cdot \;\right)}$: median value computed for the vessels in each territory that meet the criteria of diameter *D* and distance to the territory root *L.*

			Pressure [mmHg]	
District			Normotension	Hypertension
			$P_{s}$ / $P_{m}$ / $P_{d}$	$P_{s}$ / $P_{m}$ / $P_{d}$
BA			111 / 93 / 75	183 / 132 / 75
l. ICA p.			111 / 94 / 77	179 / 143 / 76
l. ICA d.			107 / 90 / 74	170 / 128 / 73
Network	Root distance	Vessel diameter	${\tilde{P}}_{\text{s}}$ / ${\tilde{P}}_{\text{m}}$ / ${\tilde{P}}_{\text{d}}$	${\tilde{P}}_{\text{s}}$ / ${\tilde{P}}_{\text{m}}$ / ${\tilde{P}}_{\text{d}}$
$T_{3}^{A}$	0cm<L≤8cm	260μm<D≤280μm	88 / 75 / 61	141 / 104 / 58
		40μm<D≤60μm	79 / 67 / 54	126 / 93 / 51
	8cm<L≤16cm	260μm<D≤280μm	69 / 60 / 48	111 / 82 / 45
		40μm<D≤60μm	67 / 58 / 46	109 / 79 / 44
	16cm<L≤24cm	260μm<D≤280μm	58 / 51 / 40	95 / 69 / 38
		40μm<D≤60μm	55 / 48 / 38	90 / 66 / 36
$T_{3}^{M}$	0cm<L≤8cm	260μm<D≤280μm	94 / 79 / 64	149 / 110 / 62
		40μm<D≤60μm	80 / 68 / 54	128 / 94 / 52
	8cm<L≤16cm	260μm<D≤280μm	76 / 65 / 52	122 / 89 / 50
		40μm<D≤60μm	71 / 60 / 48	113 / 83 / 46
	16cm<L≤24cm	260μm<D≤280μm	61 / 53 / 42	100 / 73 / 40
		40μm<D≤60μm	59 / 50 / 40	95 / 69 / 38
$T_{3}^{P}$	0cm<L≤8cm	260μm<D≤280μm	90 / 77 / 62	144 / 106 / 59
		40μm<D≤60μm	76 / 65 / 52	122 / 89 / 49
	8cm<L≤16cm	260μm<D≤280μm	67 / 58 / 46	108 / 79 / 44
		40μm<D≤60μm	62 / 53 / 42	100 / 72 / 40
	16cm<L≤24cm	260μm<D≤280μm	48 / 41 / 33	78 / 56 / 30
		40μm<D≤60μm	44 / 38 / 30	72 / 52 / 28

**Fig 7:**
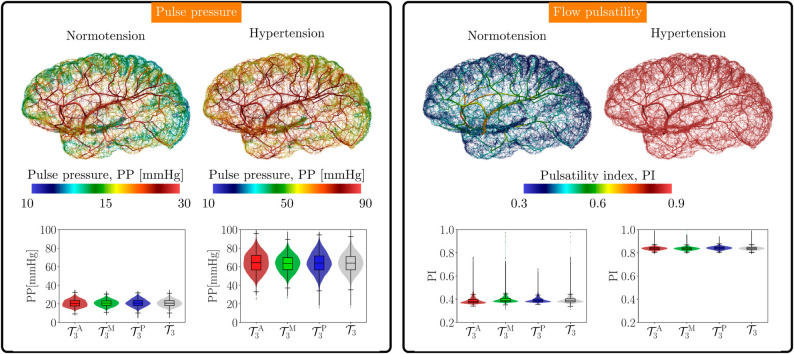
Comparison of the hemodynamics in the cerebral cortex between the normotensive and hypertensive scenarios using pulse-related indexes. Left panel: Pulse pressure across the entire cortex network as a field (top insets) and distribution of the pulse pressure in the different territories (bottom insets). Right panel: Pulsatility (flow rate-based) index across the entire cortex network as a field (top insets) and distribution of the index in the different territories (bottom insets). Statistics are shown for networks $T_{3}^{A}$ (red), $T_{3}^{M}$ (green), and $T_{3}^{P}$ (blue), and also for the entire pial network $T_{3}$ (gray), considering both scenarios, normotensive (left plots) and hypertensive (right plots).

Pulse pressure, denoted by PP, and flow pulsatility index, denoted by PI for the normotensive and hypertensive scenarios are shown in Fig 7 (in each column left: normotension, right: hypertension). Regarding the pulse pressure, we can observe a spatial variation determining reduced pulse pressure values as we move distally in the network, both in normotension and hypertension. Pulse pressure was higher in the hypertensive subject, with a wider distribution with respect to the normotensive case. The pulsatility index in the normotensive case decreased from major vessels towards the periphery, while in hypertension the values were all substantially increased, remaining within a more narrow range of variation, as can be seen in the spatial distribution and in the corresponding violin plots in Fig 7.

Table 7 presents the statistics for these two indices. Observe that these indices were roughly uniform across the three territories, and featured a narrow and also homogeneous interquartile range. The same table presents the statistics for the damping factors computed from each of those indices, namely ${\mathrm{DF}}_{\text{PP}}$ and ${\mathrm{DF}}_{\text{PI}}$, correspondingly, for the normotensive (top half) and hypertensive (bottom half) scenarios. Note that the damping factors depended on the maximum value achieved by the index on each territory, that is why the damping factor computed from the PP was roughly homogeneous across territories while the damping factor computed from the PI slightly differed for each territory due to different maxima of ${\mathrm{DF}}_{\text{PI}}$, with larger values in the MCA, followed by the ACA and then the PCA territories. From the analysis of these indices we got that the hemodynamic response in the hypertensive scenario was significantly worsened by featuring substantially increased pulse pressure (208% increase), increased pulsatility index (115% increase), and decreased damping factors (42% decrease for the pulsatility index damping factor and 5% decrease for the pulse pressure damping factor). Also, results show that the pulsatility index damping factor was a better proxy for the hypertensive scenario compared to the pulse pressure damping factor.

**Table 7:** Descriptive statistics for pulse pressure PP, the pulsatility index PI, and the damping factors obtained from these two indexes (${\mathrm{DF}}_{\text{PP}}$ and ${\mathrm{DF}}_{\text{PI}}$) in the pial vascular networks for the different territories and for the entire network in the normotensive (top half) and hypertensive (bottom half) scenarios. $\tilde{\left(\;\cdot \;\right)}$: median value, $\left[(\;\cdot \;{)}_{Q_{1}},(\;\cdot \;{)}_{Q_{3}}\right]$: lower and upper quartiles. The number of vessels $N_{V}$ is reported in Table 5.

Normotensive scenario				
Network	Pulse pressure (PP in [mmHg])		Flow rate pulsatility	
	$\tilde{PP}\;[{PP}_{Q_{1}},{PP}_{Q_{3}}]$	${\tilde{DF}}_{PP}\;[{DF}_{PP,Q_{1}},{DF}_{PP,Q_{3}}]$	$\tilde{PI}\;[{PI}_{Q_{1}},{PI}_{Q_{3}}]$	${\tilde{DF}}_{PI}\;[{DF}_{PI,Q_{1}},{DF}_{PP,Q_{3}}]$
$T_{3}^{A}$	20.5 [17.7, 23.5]	1.70 [1.48, 1.97]	0.38 [0.37, 0.40]	2.01 [1.92, 2.07]
$T_{3}^{M}$	20.8 [18.3, 23.2]	1.60 [1.43, 1.82]	0.39 [0.38, 0.41]	2.50 [2.40, 2.57]
$T_{3}^{P}$	20.8 [18.2, 23.7]	1.60 [1.41, 1.83]	0.39 [0.38, 0.40]	1.71 [1.65, 1.75]
$T_{3}$	20.7 [18.1, 23.4]	1.63 [1.44, 1.87]	0.39 [0.38, 0.40]	2.07 [1.78, 2.48]
**Hypertensive scenario**				
	**Pulse pressure (PP in [**mmHg**])**		**Flow rate pulsatility**	
	$\tilde{PP}\;[{PP}_{Q_{1}},{PP}_{Q_{3}}]$	${\tilde{DF}}_{PP}\;[{DF}_{PP,Q_{1}},{DF}_{PP,Q_{3}}]$	$\tilde{PI}\;[{PI}_{Q_{1}},{PI}_{Q_{3}}]$	${\tilde{DF}}_{PI}\;[{DF}_{PI,Q_{1}},{DF}_{PP,Q_{3}}]$
$T_{3}^{A}$	64.4 [56.5, 72.2]	1.55 [1.38, 1.77]	0.84 [0.83, 0.85]	1.18 [1.17, 1.20]
$T_{3}^{M}$	63.4 [56.8, 69.9]	1.53 [1.39, 1.71]	0.84 [0.83, 0.84]	1.40 [1.38, 1.41]
$T_{3}^{P}$	64.0 [56.6, 71.7]	1.51 [1.35, 1.71]	0.84 [0.83, 0.85]	1.12 [1.11, 1.13]
$T_{3}$	63.8 [56.7, 71.0]	1.53 [1.38, 1.73]	0.84 [0.83, 0.85]	1.20 [1.16, 1.39]

We conclude this section by reporting an analysis of the hemodynamics as we move into the depths of the vascular network $T_{3}$, as well as the hemodynamic environments encountered at the terminal outlets of network $T_{3}$. In our model, the latter represent the feeding points where perforating vessels would penetrate the pial surface into the gray and white matter to vascularize the brain tissue. Therefore, we differentiate between two different concepts: transport-level hemodynamics and perfusion-level hemodynamics.

**Fig 8:**
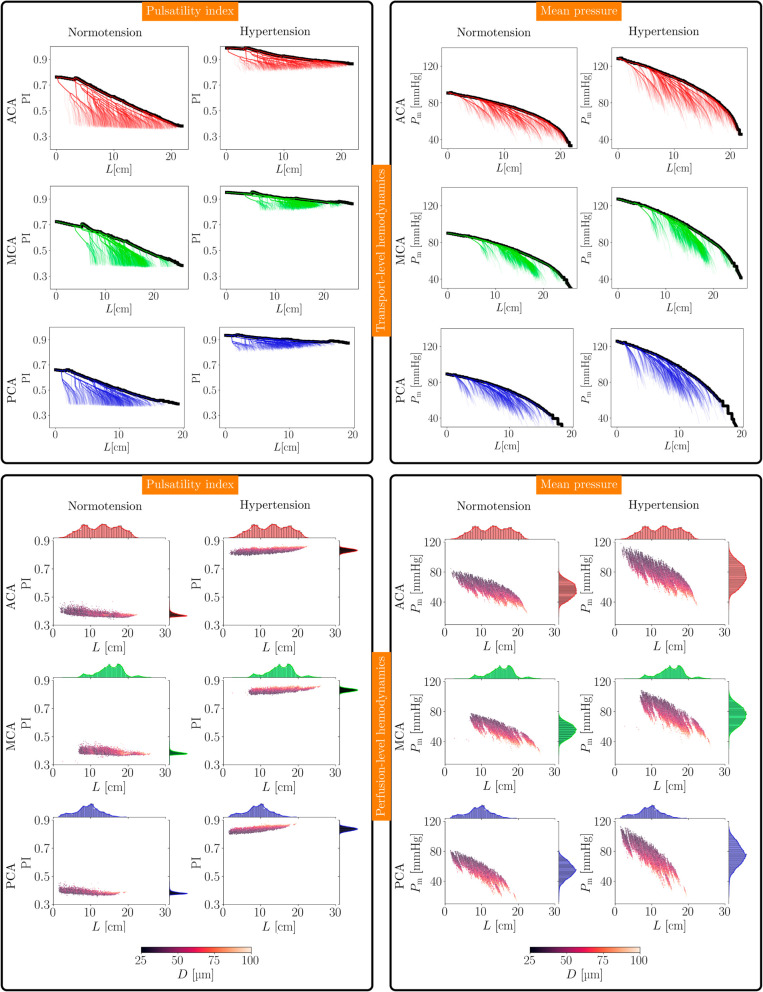
Transport-level hemodynamics (top-half) along 1000 randomly chosen vascular paths per vascular territory, and perfusion-level hemodynamics (bottom-half) defined at all terminal vessels. The transport-level quantities along the path are shown with a thick black line. Colorbar indicates vessel diameter values in the perfusion-level panels. Left panels: Pulsatility index (PI) as a function of the distance to the territory root. Right panels: Mean blood pressure as a function of the distance to the territory root. In each panel, each row corresponds to a given territory: $T_{3}^{A}$ (red), $T_{3}^{M}$ (green), and $T_{3}^{P}$ (blue). In each panel, the normotensive (left) and the hypertensive (right) scenarios are displayed.

To analyze the transport-level hemodynamics, we considered 1000 randomly chosen paths from the territory root towards the terminal outlets, for each vascular territory. Then, we analyzed the pulsatility index PI and the mean pressure $P_{\text{m}}$ along these paths. Also, we computed the maximum value of these quantities as a function of the distance to the root, denoted by *L*, resulting in the transport pulsatility index ${\text{PI}}^{t}$, and transport mean pressure $P_{\text{m}}^{t}$. Fig 8-top-half displays the transport-level analysis for the three territories (in each column left: normotension, right: hypertension). As we can see, the pulsatility index decreases almost linearly, while the mean pressure decreases nonlinearly as we travel distally within the vasculature. In the normotensive case, the transport pulsatility index declines at a rate of $\frac{d{\text{PI}}^{t}}{dx}=-1.67\;\cdot \;10^{-2}\;{cm}^{-1}$ (average of territories, linear regression model), while in the hypertensive condition that rate is $\frac{d{\text{PI}}^{t}}{dx}=-0.43\;\cdot \;10^{-2}\;{cm}^{-1}$. For the transport mean pressure, the rate of decrease is $\frac{dP_{\text{m}}^{t}}{dx}=-2.46\;mmHg/cm$ in normotension (average of territories, linear regression model), and $\frac{dP_{\text{m}}^{t}}{dx}=-3.57\;mmHg/cm$ in hypertension, characterizing a larger pressure gradient in the latter case. Inter-territorial differences in transport pressure were observed, with the PCA territory exhibiting greater transport pressure gradients and a higher rate of decay.

Concerning the perfusion-level hemodynamics, we looked into the blood flow at terminal outlets by exploring the relation between the pulsatility index ${\text{PI}}^{p}$, the mean pressure $P_{\text{m}}^{p}$, the terminal vessel diameters *D* and their distance to the corresponding territory root, denoted by *L*. Fig 8-bottom-half presents these results for the three territories (in each column left: normotension, right: hypertension). It is noteworthy that, in the normotensive scenario, the pulsatility index showed a mild decline trend as a function of the distance *L* and a less pronounced dependence on the vessel diameter *D*. In contrast, the hypertensive scenario was characterized by a mild positive trend of the pulsatility index with the distance to the territory root. The rates of change of the perfusion pulsatility index are $\frac{d{\text{PI}}^{p}}{dx}=-1.65\;\cdot \;10^{-3}\;{cm}^{-1}$ (average of territories, linear regression model) in normotension and $\frac{d{\text{PI}}^{p}}{dx}=2.28\;\cdot \;10^{-3}\;{cm}^{-1}$ in hypertension, with a greater rate of increase featured by the PCA territory. In turn, the mean blood pressure featured the expected trend as we delve towards more distant outlets for both scenarios. In normotension, the perfusion mean pressure ranges from 80mmHg to 30mmHg, with an overall drop of 50mmHg, and with a gradient of $\frac{dP_{\text{m}}^{p}}{dx}=-2.23,mmHg/cm$ (average of territories, linear regression model). In hypertension the same perfusion mean pressure dropped from 110mmHg to 40mmHg, with a difference of 70mmHg, and with a gradient of $\frac{dP_{\text{m}}^{p}}{dx}=-3.06,mmHg/cm$. Notice that, in hypertension, the increased perfusion pressure had larger impact on the proximal terminal vessels, while the distal vessels were exposed to relatively normal perfusion pressure values. Additionally, perfusion pressure exhibited greater gradients and a faster rate of decay in the PCA territory.

We can observe that there exist two concurrent hemodynamic components: heterogeneity of blood pressure across the cortex (and its associated pulse pressure) and of flow rate pulsatility. In addition, we have a differentiated manifestation of transport-level and perfusion-level hemodynamic environments. The combination of these elements determines a continuum of mechanical stimuli that affects mechanical stress in vascular walls and endothelial shear stresses differently throughout the various cortical regions. In Section Pressure gradients in the cortex, and Section Pulse pressure, flow pulsatility and the damping factor we discuss in more detail the interplay between these hemodynamic forces.

### Exploring modeling assumptions

**Fig 9:**
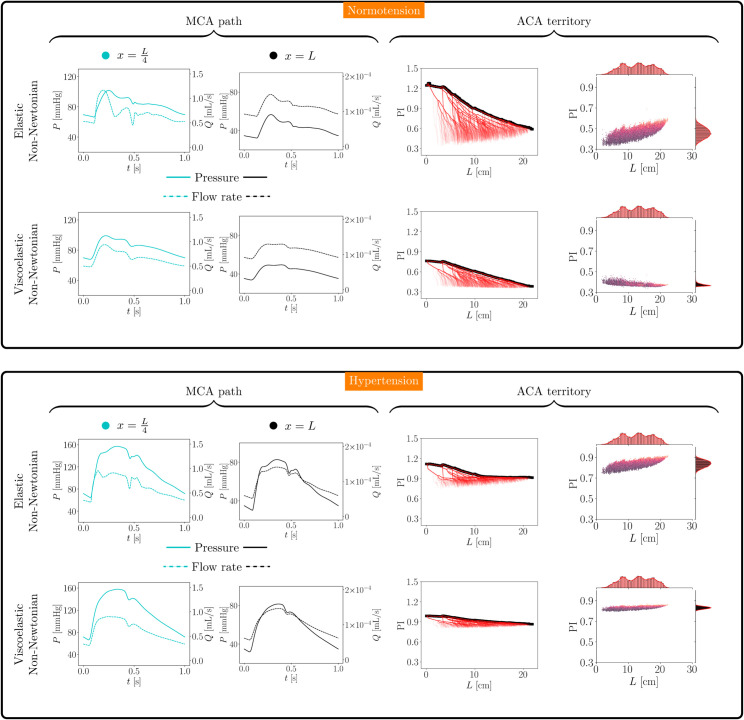
Effect of viscoelasticity in the normotensive (top panel) and hypertensive (bottom panel) scenarios. Pressure and flow rate waveforms at two different sites along the selected path through the MCA territory (left plots) and pulsatility index at transport and perfusion levels (right plots) in the ACA territory.

Concerning the constitutive behavior of the arterial wall and blood, Table 8 presents the statistics for mean pressure, pulse pressure, and pulsatility index in normotensive and hypertensive cases, considering all combinations of elastic/viscoelastic wall behavior and Newtonian/non-Newtonian blood behavior. These results correspond to the entire vascular network $T_{3}$. In addition, Fig 9 displays the comparison between elastic and viscoelastic constitutive models (case with non-Newtonian blood behavior) in terms of pressure and flow rate waveforms at two sites selected through the MCA path shown in Fig 6-central panel (corresponding to $x\in \{\frac{L}{4},L\}$, L=19.1cm), and in terms of transport-level and perfusion-level pulsatility index in the ACA territory.

**Table 8:** Descriptive statistics for mean pressure $P_{\text{m}}$, pulse pressure PP and the pulsatility index PI for the entire network in the normotensive and hypertensive scenarios considering different modeling ingredients. $\tilde{\left(\;\cdot \;\right)}$: median value, $\left[(\;\cdot \;{)}_{Q_{1}},(\;\cdot \;{)}_{Q_{3}}\right]$: lower and upper quartiles. The number of vessels is $N_{V}=234\;344$.

Normotensive scenario				
		${\tilde{P}}_{m}\;[{\tilde{P}}_{m,Q_{1}},{\tilde{P}}_{m,Q_{3}}]$	$\tilde{PP}\;[{PP}_{Q_{1}},{PP}_{Q_{3}}]$	$\tilde{PI}\;[{PI}_{Q_{1}},{PI}_{Q_{3}}]$
Newtonian	Elastic	56.7 [49.6, 63.8]	28.8 [26.2, 31.1]	0.489 [0.446, 0.531]
	Viscoelastic	56.1 [49.4, 62.8]	20.4 [17.9, 23.1]	0.386 [0.376, 0.403]
Non-Newtonian	Elastic	57.1 [50.0, 64.3]	29.0 [26.4, 31.3]	0.495 [0.452, 0.534]
	Viscoelastic	56.7 [49.8, 63.5]	20.7 [18.1, 23.4]	0.386 [0.377, 0.403]
**Hypertensive scenario**				
Newtonian	Elastic	77.3 [67.7, 87.2]	66.5 [59.1, 73.7]	0.851 [0.826, 0.871]
	Viscoelastic	76.7 [67.6, 86.1]	63.0 [56.0, 70.0]	0.838 [0.829, 0.846]
Non-Newtonian	Elastic	78.0 [68.3, 88.0]	67.1 [59.7, 74.4]	0.854 [0.831, 0.873]
	Viscoelastic	77.6 [68.2, 87.2]	63.8 [56.7, 71.0]	0.838 [0.829, 0.846]

The choice between Newtonian and non-Newtonian blood behavior has a negligible impact on the mean pressure, pulse pressure, and pulsatility index (differences of around 1%), regardless of whether the scenario involves normotension or hypertension. Differences in waveforms and the hemodynamic environment characterized by the pulsatility index are also negligible.

When comparing elastic and viscoelastic wall models, considerable differences are observed. The mean pressure is marginally smaller in the viscoelastic case, and the pulse pressure features significantly larger values in the elastic model. In normotension, differences reach 40%, and in hypertension, these differences are in the order of 6% (non-Newtonian cases). The pulsatility index also features high sensitivity in the normotensive case, with values approximately 30% larger for the elastic model, whereas in hypertension, these differences are reduced to 2% (non-Newtonian cases). The differences pointed out in the above can also be observed in the pressure and flow rate signals, where the damping effect of the viscoelastic model yields smaller pulse pressure values and flow pulsatility indexes throughout the different locations across the selected vascular path.

Notably, the pulsatility index features larger values and a wider range of variability in the elastic case at the perfusion level, i.e. at terminal sites. This trend is also present at the transport level, where the elastic case features sharper declines along the paths. These differences still exist in the hypertensive case, but are relatively smaller.

## Discussion

### Large-scale modeling of cerebral hemodynamics

Investigating realistic blood flow conditions in different regions of the brain has proven to be a challenge for the modeling community. This difficulty arises primarily from the complexity of connecting two aspects of the problem: (i) constructing vascular networks in intricate geometries and (ii) simulating pulsatile blood flow phenomena within these networks. Several notable contributions have attempted to address this issue using various strategies. Table 9 presents a comparison of the modeling strategies adopted in several contributions in this field, including the one proposed in the present work.

**Table 9:** Model ingredients adopted in the studies available in the literature.

Ref.	Vascular network	Vascular extension	Vessel diameter [μm]	Mathematical model	Flow regime	Other features
[21]	From image (rat)	$2.8\;mm^{3}$ of cortical tissue	[3.24,56.4] ^†^	0D blood flow and transport	Steady-state	Full capillary network and collaterals within cortex
[22]	From image (mouse)	$1.14-2.85\;mm^{3}$ of cortical tissue	3.8±0.3 ^‡^	0D blood flow, hematocrit and transport	Steady-state	Porous medium for surrounding tissue
[26]	From image (rat)	$2.5\;mm^{3}$ of cortical tissue	3.1±1.1 ^‡^	1D blood flow	Steady-state	Large arterioles and venules linked through porous medium
[25]	From image (human)	Large vessels in the brain	[1460,2460] ^†^	1D blood flow	Pulsatile	Large arteries of the head coupled to a porous medium representing the pial surface
[28]	Arteries from image, arterioles synthetic (human)	Entire pial surface	[50,400] ^†^	0D blood flow	Steady-state	Collaterals on mid-sized arteries plus coupling to a small-scale collateral mesh
[27]	From image (human)	Entire brain	–	3D porous medium	Steady-state	Multi-compartment model for arteries, capillaries and veins
[24]	Arteries from image, peripheral beds synthetic (mouse)	Entire brain	[3,80]^†^ ^¶^	0D blood flow and hematocrit	Steady-state	Synthetic networks provide closure between arterial and venous networks
[29]	Arteries and veins from image, peripheral beds synthetic (human)	Entire brain	[58.3, 320]^†^	0D blood flow and transport	Steady-state	Simulated magnetic resonance metrics
This work	Synthetic (human)	Entire pial surface in the left hemisphere	62.8[49.3,89.3] ^§^	1D blood flow	Pulsatile	Coupling with the entire ADAN model

†: minimum-maximum range.

‡: mean and standard deviation.

¶: extracted from cumulative distribution function.

§: median and interquartile range.

In this work, we present two major contributions from the modeling perspective: (i) the construction of a comprehensive vascular network for the entire left cerebral cortex in a human brain model, and (ii) the simulation of pulsatile 1D blood flow by coupling the ADAN model to the cerebral cortical network $T_{3}$. This was achieved by leveraging a combination of modeling tools developed by the authors in prior works [33,38,39,53–55], enabling the construction of large vascular networks in complex geometries (see Fig 5) and facilitating pulsatile simulations that account for the most salient aspects underlying the problem, including convective terms, non-linear pressure-area relationships, blood rheology, and wall viscoelasticity (see Fig 6). The following sections will discuss these two significant contributions in detail.

### Pial vascular architecture

The proposed strategy successfully replicated the complex vascular architecture of the pial surface, accounting for the intricate landscape formed by the multiple gyri and sulci present in the human cerebrum (see Fig 3), while balancing richness of complexity, anatomical constraints and computational cost [44]. Previous works applied similar strategies to generate arterial networks, and obtained results in portions of the human cerebral cortex [59], and also in the brain of rats [23,24,60]. The resulting vascular architecture obtained in this work is consistent with these previous works.

Qualitatively, the resulting vascular network closely resembles the pial vascular networks reported in the literature [61,62] (see Fig 5). Quantitatively, half of the pial vessel diameters fall within the interquartile range of [49.3,89.3]μm, with a median of 62.7μm. Regarding the terminal vessel diameters, the lower and upper quartiles are [43.4,57.6]μm with median 49.7μm (see Table 4). These ranges are consistent with the diameter of arterioles that penetrate into the cortex or form anastomoses (mostly around 40μm and 50μm) [61]. More specifically, penetrating vessels that give rise to long cortical arteries (65μm in diameter), middle cortical arteries (30μm to 60μm in diameter), and short cortical arteries (20μm in diameter) are also close to the range observed [63], especially regarding the middle intracortical arteries, which yield branches between 1.5mm and 2.0mm below the surface, a value confirmed by [40].

As previously mentioned, the vessel density of 1.2 per ${mm}^{2}$ was established in accordance with [40] (1.0 per ${mm}^{2}$), and resulted in 116 977 terminals along the 975 ${cm}^{2}$ surface area of generation (or 97 500 ${mm}^{2}$, territorial overlap included). As a matter of comparison, this is a lower value when compared to rats (8.3 per ${mm}^{2}$) [40], and the same happens when refering to the diameter of penetrating vessels [60]. Previous studies [64,65] estimated a higher number of penetrating vessels per ${mm}^{2}$ since the authors worked with primate and rodent brains. Therefore, scaling animal data to study human circulation phenomena via computational modeling must be done cautiously. The density of terminal vessels generated in the present model of the human cerebral cortex allows us to assume that the next vascularization stage involves the sprouting of penetrating arterioles that perfuse the gray and white matter tissues [40,48,65–67]. Therefore, the hemodynamic environment observed at the terminal vessels represents the mechanical stimulus to which penetrating arterioles are exposed, according to the present model.

The differences observed in inlet vessel diameters among the three territories (see Table 2, with the largest difference between the MCA and PCA at 24%) diminish as we move deeper into the pial networks (see Table 4, with the largest median difference between the MCA and ACA at 8.5%). The complete pial network presents consistent distributions of diameters, and the variation of medians from each territory to the complete network remains under 5% in all cases. The number of terminal vessels per territorial surface is a major contributor to this, since the power law for bifurcations of vessel segments [46] would scale the diameters in the binary tree down at each branching level, considering the terminal flow to be uniform as dictated by the compartmental model in the CCO algorithm [37]. Regarding this power law scaling parameter, some works [68] mention that it varies along the cortex, from 3.61 in the larger arteries, down to 2.73 when penetrating the cortex. Here, this parameter was considered to be constant and equal to 3.0, which corresponds to the case of uniform endothelial shear stress [46,49]. Previous settings used for the generation of the cerebral cortical network, considering the power law parameter of 3.5, rendered vessels with substantially larger diameters when targeting the terminal density of 1.2 terminal vessels per ${mm}^{2}$, which was not aligned with the literature [61]. Furthermore, according to [69], the arborizations that perfuse the cortical tissue do not follow a power law model, but are better represented by a stem-crown model. That study is of the utmost relevance towards the construction of vessel branches located at more distal locations in our model.

Notably, despite significant differences in the geometric features and covered areas of the ACA, MCA, and PCA territories, the vessel lengths remain significantly consistent across the three territories (see Table 4, where the largest median difference between the ACA and MCA territories is 1%).

### Pressure gradients in the cortex

In a previous study [32], we provided compelling evidence for the presence of significant pressure gradients within the human cerebral cortex. In that research, we used a simplified model of the peripheral vascular beds located at the base of the brain–specifically, distal to the lenticulostriate artery–and over the convexity, particularly distal to the posterior parietal branch of the middle cerebral artery. These findings have impacted the analysis and interpretation of various conditions associated with small vessel disease and neurodegenerative disorders. Importantly, understanding the causes of neurodegenerative conditions related to blood flow in light of the heterogeneities encountered in cerebral hemodynamics has opened new research avenues in this field. As a result of that contribution, there has been increased awareness regarding the appropriateness of applying uniform blood pressure targets across the general population [70–73].

The ambibaric brain theory has offered a new evolutionary perspective on the cerebral circulation, featuring essential physiological, pathophysiological, and clinical implications [31]. From the experiments performed in the present study, we observe that the pressure levels in peripheral vascular beds vary depending upon the specific region of the cortex being examined. Regions more distant from the Circle of Willis exhibit the lowest pressure levels, regardless of the specific territory. In contrast, areas closer to the base of the brain, even when part of the cortex, display higher pressure levels. As a rule of thumb, the greater the vascular distance traveled by blood, the lower the recorded pressure (see Figs 6 and 8-top right panel). It is important to note that this model represents the cortical vasculature down to the terminal vessels that stand for the arterioles that penetrate the gray/white matter tissues. The pressure levels and flow pulse experienced by these distal columnar arborizations, which derive from the arterioles and supply the gray and white matter, differ significantly based on the location of the terminal vessels. Therefore, the findings reported in this work not only support the ambibaric brain theory, but also illustrate a continuum of pressure levels from the base of the brain to the deepest regions of the cortex. Additionally, the compartmentalization of these deeper cortical areas and the differentiation of hemodynamic environments at both the transport and perfusion levels bring relatively novel aspects that deserve further investigation. (see Fig 8-right panels).

From the results reported in this work, we note that the range of pressure levels is wider in hypertension with respect to the one observed in normotension. Systolic hypertension is characterized by elevated systolic pressure, moderately increased mean pressure, and slightly decreased diastolic pressure, leading to a higher arterial pulse pressure (see Table 6). Pressure gradients experienced by the cerebral vasculature are greater in a hypertensive state, which creates more heterogeneous mechanical stimuli across the cortex, thereby promoting remodeling in a spatially differentiated manner (see Fig 8-right panels). Proximal territories exposed to higher pressure and pulse pressure may exhibit a different remodeling mechanism than distal territories [74,75]. This region-dependent maladaptation may contribute to the acceleration of detrimental conditions affecting the brain.

Moreover, according to the reported simulations, the lowest pressure values in the cortical network change little, with differences around 10mmHg when comparing normotensive and hypertensive conditions (see Table 6 and Fig 8-bottom right panel). This suggests that any treatment aimed at reducing central arterial pressure might exacerbate the reduction of diastolic blood pressure at the smallest arteriolar vessels that supply the most distal regions of the cortex [71,72]. Although the cerebrovascular compliance provides some protective effects for these distal areas regarding pressure, pulsatile conditions are significantly worsened in hypertension at both the transport and perfusion levels (see Figs 7 and 8-left panels). This combination of low blood pressure with high pulsatility (see next section) creates a harmful environment that may trigger specific adaptations in the distal regions of the hypertensive brain, which are different from those encountered in proximal locations [76].

### Pulse pressure, flow pulsatility and the damping factor

The hypertensive condition establishes a pathological interaction between macro- and microvascular vessels. The combined effects of these adaptations significantly alter the shape of the pressure waveform as it travels from the aortic root to the periphery. This alteration provides a strong mechanical stimulus that affects local flow conditions, including endothelial shear stresses and the mass transfer of substances between blood flow and surrounding tissues in smaller vessels. Elevated pressure, pulse pressure, and flow pulsatility create deleterious hemodynamic conditions that extend from the central vessels to the microcirculation [77].

In addition to the individual values of systolic and diastolic blood pressure, pulse pressure is an important surrogate of arterial stiffness, a key aspect of the aging cardiovascular system in both normotensive and hypertensive patients, which can result in microvascular damage [78]. Furthermore, hypertension may accelerate the process of arterial stiffening through a positive feedback cycle, which can worsen hypertension itself [79]. It has also been well established that there exists a relationship between the flow pulsatility index and various brain diseases [80], as well as the impact of hypertensive conditions on this index [81]. More recently, Arts et al. [82] explored the relationship between pulsations in large arteries and how these pulsations are transmitted to small arteries and the microcirculation, through the concept of cerebrovascular damping. Their findings indicated that in healthy individuals, there is a damping effect–quantified by the damping factor as defined here–on flow pulsatility when comparing the root of the MCA territory to small cerebral perforating arteries. Specifically, the pulsatility and damping factors for healthy individuals were reported to be 0.40±0.14 and 2.1±0.81 for vessels around the basal ganglia, and 0.42±0.13 and 2.0±0.93 for vessels around the semi-oval center. These biomarkers are intended to describe the transfer of energy from central circulation to peripheral vascular beds. In this study, we directly assessed the hemodynamics in the deeper regions of the cerebral vasculature and estimated changes in both the pulsatility index and pulse pressure over the convexity. It is important to note that our simulations rendered maximum values of the pulsatility index, and of the pulse pressure (see (14)) in the neighborhood of the root of each vascular territory (see Fig 7). Hence, we obtained a damping factor for the pulsatility index (median [IQR]) of 2.07[1.78,2.48] in normotensive individuals, compared to 1.20[1.16,1.39] in hypertensive patients (see Table 7). These results are also consistent with those observed by Van Den Kerkhof et al., [83], who utilized magnetic resonance imaging to investigate healthy and hypertensive subjects. That study also showed a positive correlation between the pulsatility index and arterial pressure, while the damping factor exhibited a negative correlation with arterial pressure. In another study by the same research group [84], they found that a reduced damping factor–calculated using the pulsatility index of the middle cerebral artery and the lenticulostriate artery–was significantly associated with the presence of perivascular spaces in the basal ganglia, a condition commonly seen in small vessel disease [85]. Furthermore, this correlation was even stronger in hypertensive patients.

Zarrinkoob et al. [86] explored the damping effect of the cerebral vasculature on the flow pulsatility from the proximal to the distal cerebral arteries. The research involved both healthy young and elderly subjects, while also examining the relationship to aortic stiffness. Their findings revealed that the cerebral vasculature in younger individuals exhibits a more pronounced damping effect on flow pulsatility compared to older individuals. The damping factors varied across different arterial territories. In that study, these factors were specifically related to pulsatility indexes of the internal carotid artery and the vertebral artery. The damping factor values recorded were 1.31±0.20 for young subjects compared to 1.16±0.20 for elderly subjects for the distal middle cerebral artery, 1.48±0.25 for young versus 1.26±0.22 for elderly subjects for the distal anterior cerebral artery, and 1.69±0.38 for young subjects compared to 1.32±0.20 for elderly subjects for the posterior cerebral artery. The combination of increased pulsatile flow and reduced damping factors supports the theory of pulse wave encephalopathy (PWE) [87], which suggests that higher pulsatile flow reaching more distal arterial segments can lead to damage in the cerebral microvasculature. Additionally, a new metric called the pulsatility transmission function was proposed in [88] to classify the risk of PWE more effectively. This new index aims to provide a better understanding of cerebral hemodynamics at the organ level using spatially distributed data obtained from magnetic resonance imaging.

The results obtained in the present study provide valuable insights that build on previous research. The pulse pressure affecting peripheral vessels is lower than that at the base of the brain (see Table 6 and Fig 7), with this damping effect being only mildly stronger in normotensive conditions compared to hypertensive ones. The pulsatility index is a better indicator of hypertensive states, showing not only higher and more uniform values across the convexity but also a significantly reduced damping effect (see again Fig 7). Typically, maximum pulsatility occurs in the root vessels. At the transport level, both the pulse pressure and the pulsatility index decrease as we move towards more distal locations in the vascular network in both normotensive and hypertensive conditions (see Fig 8-top left panel). However, the situation is different at the perfusion level. While pressure continues to decrease when moving distally for normotensive and hypertensive cases, the pulsatility index features an inverted trend in hypertension compared to normotension, and with values considerably higher, which pose detrimental conditions in the hypertensive state (see Fig 8-left panels). Therefore, the vascular adaptations that occur in the cerebral vasculature depend on the location of the vessel [89]. This suggests that, in normotensive cases, blood flow is more uniform along the cardiac cycle, and so endothelial shear stresses. In contrast, hypertensive conditions are characterized by stiffened vascular walls, and more fluctuating shear stresses, which can lead to increased flow-induced cyclic fatigue in the endothelial cells, affecting endothelial permeability, mass transport, and cerebrovascular reactivity, among other functional properties of the brain [90,91].

### Pathophysiology of neurodegenerative diseases

Diseases of the nervous system have become the leading causes of disability-adjusted life years (DALYS). Among these, stroke accounts for 52% and dementia for 9% [92]. The major neurodegenerative condition is dementia, and among dementias what is labeled as “Alzheimer’s Disease” is the most common. However, the typical patient with this diagnosis harbours up to 8 different pathologies [93].

Moreover, all major dementias have a vascular component, ranging from 61% in frontotemporal dementia and up to 83% in Alzheimer’s disease [94]. Vascular and neurodegenerative conditions not only occur together but interact at all levels, including the microcirculation [95].

The concept of the ambibaric brain offers a framework to interpret brain lesions; for example, it explains the mechanism of the common association of apathy, gait disorders and executive dysfunction (the AGED triad) [96].

In light of these findings, the present contribution provides a mechanistic substratum based on first principles to allow us to quantify the hemodynamic environment and its potential association with the onset and progress of these conditions. According to the findings reported in this study, the heterogeneity in the mechanical stimuli (see Figs 6, 7 and 8) that take place over the pial surface should draw attention to their spatial dependence for the explanation of disease etiology.

### Impact of modeling assumptions

In this study, we explored the constitutive assumptions regarding blood behavior (Newtonian/non-Newtonian) and vascular wall behavior (elastic/viscoelastic). We observed that the choice of blood behavior did not impact the model predictions. Indeed, vessel diameters in the CCO vascular network are primarily within the range where the viscosity, as predicted by the non-Newtonian model, does not substantially differ from the Newtonian case. Simulations involving a larger number of smaller vessels may be more sensitive to the blood constitutive model (see Table 8).

Regarding the comparison between elastic and viscoelastic wall models, we have observed that differences are substantial, mainly in terms of the oscillatory components of pressure and flow rate that are responsible for the pulse pressure, the pulsatility index, and the overall waveform signature (see Fig 9). In terms of flow pulsatility, the elastic wall model predicted larger values from the root to the terminal sites, with a wider distribution of values and a positive trend between the outlet distance to the root and the pulsatility index, something that is inverted in the viscoelastic case. Notably, this would explain the modification of the rate of change in the pulsatility index observed in hypertension because, in that scenario, the elastic component is increased compared to the viscoelastic case. Therefore, viscoelasticity is key in the damping of flow pulsatility towards the more distant sites of the cortical circulation.

### Limitations

We have adopted a simplified approach to model hypertension by adjusting model parameters in a manner similar to that used in previous studies in this field. Additionally, we have constructed a single vascular model, which restricts our analysis to one cerebrovascular architecture. We have not included blood flow control mechanisms, as we considered that the characterization of the hypertensive scenario already takes into account the compensatory adjustments of the cardiovascular system under such pathological condition. Peripheral beds were modeled with Windkessel elements, and no venous blood vessels were considered in the network. Also, the extensive network was built only for the left hemisphere, while the right hemisphere topology remained as in the original ADAN model, and no collateral connections were included in the proposed model, assuming that the cortex vascular network is completely patent. If we were to include obstructive lesions, as seen in other contributions, it would be necessary to incorporate inter-hemispheric and intra-hemispheric collateral connections that would become activated, due to intense pressure gradients, in such pathological scenarios. In addition, the bifurcation power law parameter was considered constant throughout stages in this study, as discussed in Section Pial vascular architecture. An approach that varies this parameter along the levels of bifurcation would be more suitable, especially when constructing the penetrating arborizations that vascularize the gray and white matter tissues.

## Final remarks

In this study, we proposed a computational modeling strategy to investigate the hemodynamics in the pial surface that supplies human brain’s cortex.

First, we exploited advanced computational tools to automate the creation of a vascular network based on an anatomically consistent geometric representation of the pial surface in a patient-specific model of the human brain. This process achieved a terminal vessel density of $1\;vessel/mm^{2}$, which corresponds to the density of penetrating arterioles that supply the underlying gray and white matter tissues. The resulting model includes 234 344 vessel segments with a total cumulative length of 171.1m and an intravascular volume of $2.88\;cm^{3}$. The median [IQR] vessel diameter in the vacular network is 62.7[49.3,89.3]μm.

Second, we presented blood flow simulations that achieved an unprecedented level of detail by coupling the pial vascular network to the ADAN model. This allowed us to provide estimates of hemodynamic phenomena ranging from the large arteries down to the arterioles that penetrate the cortical gray and white matter. Under normotensive conditions, the median pressure and flow rate values are 56.7[49.8,63.5]mmHg and $6.9\;[3.4,19.9]\;\;\times \;10^{-5}ml/s$, respectively.

We have successfully modeled the distribution of blood flow-related quantities, including systolic, diastolic, and mean blood pressure, pulse pressure, pulsatility index, and associated damping factors in both normotensive and hypertensive conditions. As expected, systolic pressure is significantly elevated in hypertension, while we noted a slight decrease in diastolic pressure. For reference, while normotensive (hypertensive) readings in the brachial artery are 111/75mmHg (183/75mmHg), these values at the distal internal carotid artery pressure are 107/74mmHg (170/73mmHg). Correspondingly, in the smaller vessels of the distal anterior cerebral artery territories, the median values are 55/38mmHg (90/36mmHg), while being 59/40mmHg (95/38mmHg) in the distal middle cerebral artery territories, and 44/30mmHg (72/28mmHg) in the distal posterior cerebral artery territories. Thus, the posterior cerebral territories are more susceptible to hypotension in this model.

By examining the pressure levels at the terminal vessels, we observed that the spatial pressure gradients over the convexity in normotension were limited to 50mmHg, while in hypertension, these gradients reached up to 70mmHg. These gradients can be explained by the cumulative vascular distance that blood must travel before reaching its final destination in the various territories on the pial surface before penetrating into the gray and white matter tissues. Notably, the farther a pial vessel is from the Circle of Willis, the lower its pressure level is. Additionally, the diameter of the vessels is not as critical as the path length in determining blood systolic and diastolic pressures.

Flow-related indices aligned well with values reported in specialized literature. When comparing hypertension to normotension, the median [IQR] pulse pressure resulted 20.7[18.1,23.4]mmHg for normotension and 63.8[56.7,71.0]mmHg for hypertension. The pulsatility index was 0.84[0.83,0.85] versus 0.39[0.38,0.40], and the damping factor was 1.20[1.16,1.39] versus 2.07[1.78,2.48]. Increased pulsatility and reduced damping factors promote a highly fluctuating shear stress on endothelial cells, which may lead to structural fatigue and dysfunction of the endothelial layer.

Finally, we also concluded that viscoelasticity is a key component in the conformation of pressure and flow rate waveform signatures, having a significant impact on the characterization of pulse pressure and the flow pulsatility index at the transport level down to the perfusion level.
